# Essential Oils Biofilm Modulation Activity, Chemical and Machine Learning Analysis. Application on *Staphylococcus aureus* Isolates from Cystic Fibrosis Patients

**DOI:** 10.3390/ijms21239258

**Published:** 2020-12-04

**Authors:** Rosanna Papa, Stefania Garzoli, Gianluca Vrenna, Manuela Sabatino, Filippo Sapienza, Michela Relucenti, Orlando Donfrancesco, Ersilia Vita Fiscarelli, Marco Artini, Laura Selan, Rino Ragno

**Affiliations:** 1 Department of Public Health and Infectious Diseases, Sapienza University, p.le Aldo Moro 5, 00185 Rome, Italy; rosanna.papa@uniroma1.it (R.P.); gianluca.vrenna@uniroma1.it (G.V.); marco.artini@uniroma1.it (M.A.); 2 Department of Drug Chemistry and Technology, Sapienza University, p.le Aldo Moro 5, 00185 Rome, Italy; stefania.garzoli@uniroma1.it (S.G.); manuela.sabatino@uniroma1.it (M.S.); filosapi@gmail.com (F.S.); 3 Rome Center for Molecular Design, Department of Drug Chemistry and Technology, Sapienza University, p.le Aldo Moro 5, 00185 Rome, Italy; 4 Department of Anatomy, Histology, Forensic Medicine and Orthopaedics, Sapienza University of Rome, via Alfonso Borelli 50, 00161 Rome, Italy; michela.relucenti@uniroma1.it (M.R.); orlando.donfrancesco@uniroma1.it (O.D.); 5 Paediatric and Laboratory Department, Children’s Hospital and Institure Research Bambino Gesù, 00165 Rome, Italy; evita.fiscarelli@opbg.net

**Keywords:** essential oil, GC-MS analysis, machine learning, classification algorithms, scanning electron microscopy, cystic fibrosis, antibacterial, antibiofilm, *Staphylococcus aureus*

## Abstract

Bacterial biofilm plays a pivotal role in chronic *Staphylococcus aureus* (*S. aureus*) infection and its inhibition may represent an important strategy to develop novel therapeutic agents. The scientific community is continuously searching for natural and “green alternatives” to chemotherapeutic drugs, including essential oils (EOs), assuming the latter not able to select resistant strains, likely due to their multicomponent nature and, hence, multitarget action. Here it is reported the biofilm production modulation exerted by 61 EOs, also investigated for their antibacterial activity on *S. aureus* strains, including reference and cystic fibrosis patients’ isolated strains. The EOs biofilm modulation was assessed by Christensen method on five *S. aureus* strains. Chemical composition, investigated by GC/MS analysis, of the tested EOs allowed a correlation between biofilm modulation potency and putative active components by means of machine learning algorithms application. Some EOs inhibited biofilm growth at 1.00% concentration, although lower concentrations revealed different biological profile. Experimental data led to select antibiofilm EOs based on their ability to inhibit *S. aureus* biofilm growth, which were characterized for their ability to alter the biofilm organization by means of SEM studies.

## 1. Introduction

Cystic fibrosis (CF) is a hereditary disease that affects the normal function of epithelial cells, especially in the lungs and digestive system, causing incremental disability. Recurrent and chronic respiratory tract infections in CF patients result in progressive lung damage representing the primary cause of morbidity and mortality. *Staphylococcus aureus* (*S. aureus*) is one of the earliest bacteria detected in infants and children affected by CF [1]. *S. aureus* is the prevalent microorganism in CF children with a maximum prevalence occurring in ages of 11–15 years [2]. The increasing diffusion of methicillin resistant *S. aureus* (MRSA) strains in the last 10 years has gained the attention of the scientific community [3]. Statistical studies revealed that CF patients infected by MRSA in the respiratory tract show worse clinical outcomes [4,5]. For this reason, from a young age, CF patients are treated with antimicrobial drugs to control lung infections. 

*S. aureus* possesses a variety of virulence factors including the ability to form biofilm, which plays a pivotal role in chronic infections. Indeed, chronic *S. aureus* infection in CF patients’ lung, sustained in the biofilm phenotype, is associated with in vitro antibiotic resistance [6]. Biofilm mode of growth does occur in MSSA and MRSA regardless of genetic background [7]. The highest prevalence of methicillin resistant *S. aureus* (MRSA) arises in individuals from 10 to 30 years old, while MSSA are prevalent in patients younger than 10 (Cystic Fibrosis Foundation, 2017).

Despite the growing evidence that lung infections in CF patients are sustained by biofilm, antibiotics effectiveness is mostly evaluated in planktonic cell assays. This produces misleading results, as a bacterial strain can be sensitive to an antibiotic in vitro and assume resistance in vivo due to biofilm formation in CF lung airways. 

A negative modulation or a complete inhibition of biofilm formation may represent an important strategy for infection control and might be considered as a major target for the development of novel therapeutic agents [8]. Hence, there is a need to develop innovative approaches that lower or block biofilm formation without affecting bacterial vitality, avoiding the appearance of escape mutants [9,10]. The use of anti-biofilm compounds could enhance the effectiveness of conventional therapies [11,12], particularly in chronic infections; this could represent a suitable approach to treat CF patients’ infections. A complex challenge, therefore, remains to prevent biofilm formation and to try to disrupt the existing biofilm.

During the past decade, the scientific community has oriented itself towards “green alternatives” such as natural compounds, including essential oils (EOs) and their main chemical components [13]. Many bioactive compounds extracted from plants are known to exert antimicrobial properties as evinced by a large use in traditional medicine [14]. EOs are complex mixtures composed of different classes of compounds and have been empirically used for centuries to treat upper respiratory tract infections such as pharyngitis, sinusitis and bronchitis [15]. Bacteria hardly develop resistance to multi-component treatments as EOs, likely due to their multitarget actions [16]. 

Recently, a series of 90 EOs were investigated for their abilities to modulate the bacterial biofilm production in cultures of different *S. aureus*, *S. epidermidis* and *Pseudomonas aeruginosa* strains [17,18]. Interestingly some EOs were able to strongly inhibit biofilm production at not antimicrobial concentrations, whereas some other EOs showed to strongly stimulate biofilm production. To shed light on the possible role of EOs’ chemical components, machine learning (ML) algorithms were applied to develop classification models. Analysis of the ML models indicated the chemical components mainly responsible for bacterial biofilm production inhibition or stimulation. In a later report, ML-based clustering was used to develop a convergent microbiological protocol in which 61 EOs were evaluated on 40 clinical isolated *S. aureus* and *P. aeruginosa* strains [19]. In that study, three essential oils showed to be able to impair bacteria vitality in all tested strains. Based on these results, it is herein reported the modulation of bacterial biofilm production of *S. aureus* strains isolated from CF patients of previously characterized EOs. The effect of the most promising EOs on biofilm formation is also observed by SEM analysis on selected *S. aureus* reference and clinical strains. Furthermore, continuing the investigation on EOs [20], in a multidisciplinary approach [21,22] and in agreement with the recent reports [23,24,25], ML is used to shed light on the EOs chemical components likely responsible to positively or negatively modulate the bacteria biofilm formation.

## 2. Results

### 2.1. Biofilm Production Modulation by EOs at Selected Fixed Concentrations

The EOs’ ability to modulate biofilm production by *S. aureus* strains was evaluated at two different concentrations. In particular, the concentration of 1.00% *v*/*v* was chosen on the basis of a previous report [19]. At this concentration, the antimicrobial activity of the 61 EOs listed in Table SM4 was evaluated, and inactive EOs were investigated for their ability in modulating the biofilm production (Table SM5). A second sub-antimicrobial concentration of 0.05% *v*/*v*, selected in agreement with previous studies [17,18], was also used to evaluate the EOs biofilm modulation at low concentration. At both tested concentrations (1.00% *v*/*v* and 0.05% *v*/*v*) the biofilm production was compared to that of untreated bacteria (Tables SM6 and SM7). 

### 2.2. Quantitative Analysis of Biofilm Production by S. aureus Strains Treated with Selected EOs 

**Figure 1:**
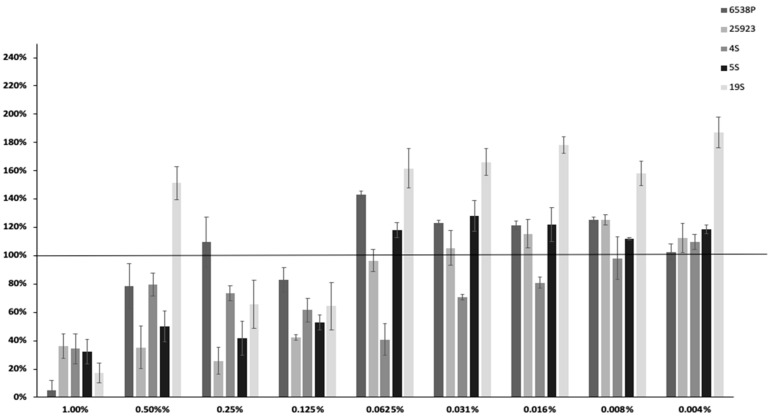
Dose-dependent effect of EO45 on different clinical and reference strains starting from a concentration of 1% to 0.004% *v*/*v*. In the ordinate axis is reported the percentage of bacterial biofilm production. Data are expressed as percentage of residual biofilm after the treatment in comparison with untreated one. Each data point is composed of four independent experiments each performed at least in three replicates.

**Figure 2:**
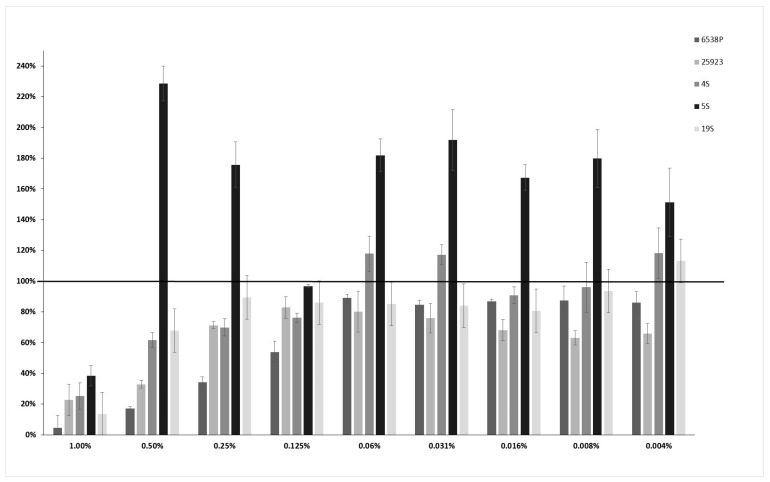
Dose-dependent effect of EO58 on different clinical and reference strains starting from a concentration of 1% *v*/*v* to 0.004% *v*/*v* biofilm. In the ordinate axis is reported the percentage of bacterial biofilm production. Data are expressed as percentage of residual biofilm after the treatment in comparison with untreated one. Each data point is composed of 4 independent experiments each performed at least in 3-replicates.

EO45 and EO58 were selected as they showed to strongly reduce biofilm formation more than 60% in all tested strains (Table SM6) at concentration of 1.00% *v*/*v*. EO45 and EO58 were further analyzed to search for a possible dose-dependent effect in the 5 tested *S. aureus* clinical and reference strains (Figure 1 and Figure 2). The dose-dependent effect of EO45 was evaluated starting from a concentration of 1.00% *v*/*v* to 0.004% *v*/*v*. The inhibition by EO45 was confirmed up to a dilution of 0.125% *v*/*v* with some exceptions (except for strain 19S at 0.50% *v*/*v* and strain 6538P at 0.25% *v*/*v*); despite different phenotypic features of the strains, the inhibition of biofilm formation did not show a dose-dependent response (Figure 1), as already observed on *P. aeruginosa* [17]. On the contrary, at lower concentrations the EO45 showed no modulation or could even suggest a tendency to stimulate biofilm production.

Differently from EO45, for EO58 an initial dose-dependent negative modulation of biofilm production was observed against 6538P, 25293, 4S and 19S strains in a concentration range between 1.00% and 0.06% *v*/*v*, while it did not induce biofilm formation at lower concentrations, in absence of any recognizable pattern (Figure 2). In the case of 5S strain, EO58 performed prevalently as a biofilm production enhancer.

### 2.3. SEM Observation of Eos Action on Biofilm Formation

**Figure 3:**
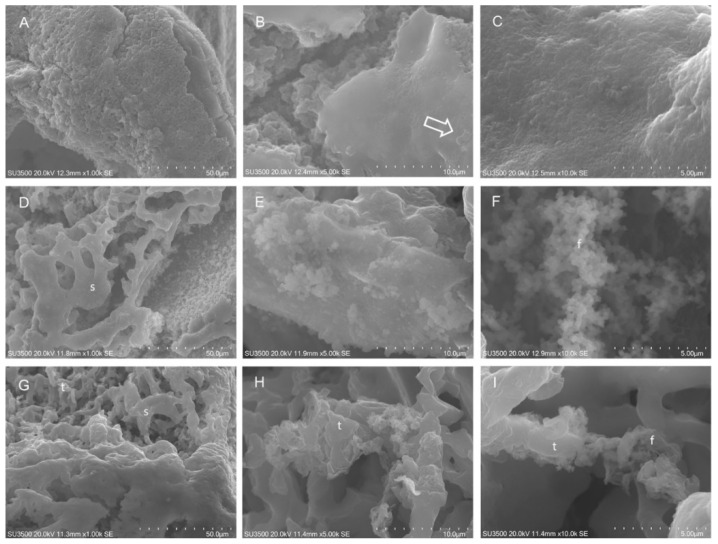
SEM analysis on *S. aureus* ATCC 25923. Untreated *S. aureus* 25923: (**A**) SEM, 1000×. Compact biofilm surface. (**B**) SEM, 5000×. Spongy structure beneath the compact area, on the surface bacterial cells were visible (arrow). (**C**) SEM, 10,000×. At very high magnification, the smooth aspect of compact area is shown. *S. aureus* 25923 after exposure to EO45 1.00% *v*/*v*: (**D**) SEM, 1000×. Skeletonized aspect of dissolved compact areas. (**E**) SEM, 5000×. Bush-like floccular aggregates on compact area surface. (**F**) SEM, 10,000×. EPS is melted in very fine filaments. *S. aureus* 25923 after exposure to EO58 1.00% *v*/*v*: (**G**) SEM, 1000×. Compact areas appeared skeletonized by oil action, trabeculae were thinned. (**H**) SEM, 5000×. At high magnification, erosion of trabeculae in spongy areas was evident; (**I**) SEM, 10000×. At very high magnification, trabeculae flaking and disintegration in fine filaments were visible. t: trabecula; f: filaments; s: sponge.

Biofilm effects of EO45 and EO58 were also investigated by SEM analysis. Based on dose-dependent analysis results (Figure 1 and Figure 2), EO45 was explored on *S. aureus* 5S (biofilm inhibition higher than 40% at all tested concentrations) while EO58 on *S. aureus* 4S. Both EOs were also separately tested on reference strain *S. aureus* 25923 (Figure 3). Imaging of the untreated biofilm from *S. aureus* 25923 provided the expected morphology with compact and smooth surfaces, and an inner spongy structure (panels A and B) even at very high magnification (panel C). *S. aureus* 25923 biofilm treated with EO45, revealed the compact part to be broken down and modifications recalling the spongy part. At high magnifications was recognized formation of bush-like floccular aggregates (panel E). At increased magnification, EPS disintegration in fine filaments was visible (panel F). Treatment with EO58 induced very similar effects, breakup of compact areas (panel G), erosion of trabeculae in spongy areas and EPS flaking and disintegration (panel I). 

**Figure 4:**
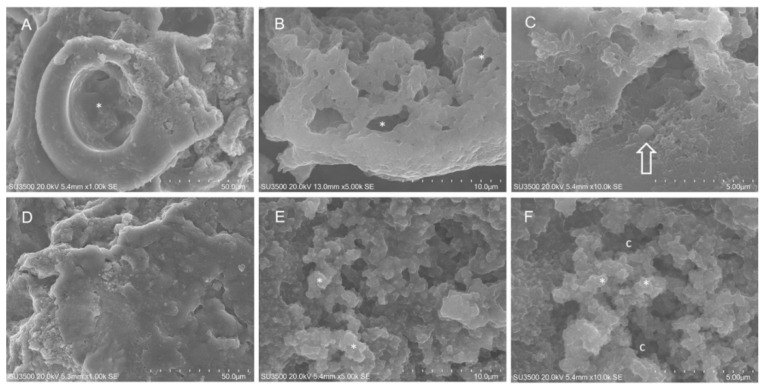
SEM analysis on *S. aureus* 4S. Untreated *S. aureus* 4S: (**A**) SEM, 1000×. At this magnification a macro channel is visible (asterisk), its walls were made of smooth, compact and dense biofilm, in the right part of the picture spongy biofilm is present. (**B**) SEM, 5000×. Higher magnification of microchannel system (asterisk), it appeared as a three-dimensional network with irregular meshes. (**C**) SEM, 10,000×. Very high magnification showed bacterial cells partially embedded in EPS (arrow). *S. aureus* 4S after exposure to EO58 1.00% *v*/*v*: (**D**) SEM, 1000×. Smooth surface of compact biofilm areas has become rough. (**E**) SEM, 5000×. Largest and superficial trabeculae were melted by oil action, the innermost and smallest were still present (asterisk). (**F**) SEM, 10,000×. Trabeculae breakdown caused stumps retraction and matrix thickening (asterisk), microchannels merging resulted in large caves that opened up. c: caves.

SEM analysis was also performed on untreated 4S (Figure 4). The *S. aureus* 4S strain secreted a dense biofilm, in which macrochannels (Figure 4 panel A) 35–40 µm in diameter were developed. Biofilm showed compact and spongy areas (Figure 4 panels A–C), in which a network of microchannels were established displaying comparable sizes. Some bacterial cells were visible and partially embedded in the extracellular polymeric substance (EPS) (Figure 4 panel C). EO58 exerted on *S. aureus* 4S biofilm a disruptive action. The smooth surface of the denser areas acquired an irregularly wrinkled aspect (Figure 4 panels D–E). The trabeculae, which formed the microchannel system, thinned out or disappeared. EO action melted the largest and superficial trabeculae, while the innermost and smallest were preserved (Figure 4 panel E). A decrease in biofilm compactness was evident, due to merging of microchannels into large spaces (caves, c). Trabeculae stumps retracted and their thickened extremities were visible (Figure 4 panels E–F).

**Figure 5:**
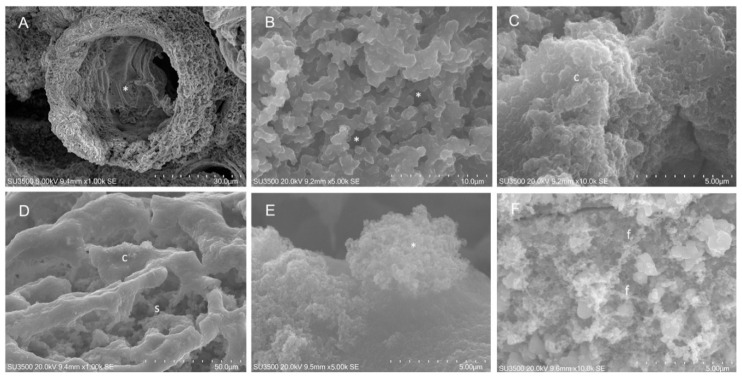
SEM analysis on *S. aureus* 5S. Untreated *S. aureus* 5S: (**A**) SEM, 1000×. Biofilm showed microchannels (asterisk). Biofilm surface was compact and rough; (**B**) SEM, 5000×. Inner areas with spongy structure. (**C**) SEM, 10,000×. Sometimes, inner dense areas with compact arrangement were observed. *S. aureus* 5S after exposure to EO45 1.00% *v*/*v*: (**D**) SEM, 1000×. Compact areas flakes off, allowing inner spongy structure to appear. (**E**) SEM, 5000×. High magnification showed signs of EPS disintegration in the way of a bush-like floccular aggregate (asterisk). (**F**) SEM, 10,000×. Very high magnification shows trabeculae flaking in fine filaments. f: filaments; s: sponge; c: caves.

SEM analysis on *S. aureus* 5S (Figure 5) revealed that untreated bacteria biofilm appeared pierced by numerous macrochannels of 10–15 µm in diameter, whose surface was compact and grossly rough for the presence of large globular masses of EPS. The inner aspect was spongy and was formed by an intricate three-dimensional network of short EPS trabeculae, among which a microchannel system developed. In some inner areas, instead of a trabecular system, a denser EPS arrangement was observed. Treatment with EO45 had a remarkable effect on *S. aureus* 5S biofilm as the compact surface appeared deconstructed, displaying inner spongy areas. Increasing magnification allowed detailed observation of EPS melting revealing on compact areas surface, bush-like floccular aggregates. Higher magnifications revealed the dispersion of EPS components in bush-like floccular aggregates, which appear dispersed in a cloud of very fine filaments. Fraying of spongy EPS trabeculae was evident.

### 2.4. Essential Oil Chemical Composition

GC/MS analyses were carried out on the 61 EOs (Table SM4), revealing a total of 239 chemical components differently distributed among EOs (Tables SM8 and SM19). Herein, are report the composition of the above selected EO45 and EO58, revealing two different chemical profiles (Table 1). Details on other EOs are available upon request.

### 2.5. Machine Learning Binary Classification

#### 2.5.1. Datasets

Considering the antimicrobial activity (Table SM5), the biofilm production investigations (Tables SM6 and SM7) and the five S. aureus strains, a total of 15 different initial datasets were loaded into a Pandas dataframe. Each dataset was composed by a data matrix of 61 rows (essential oil samples) and 240 columns (one bioactivity and 239 chemical components). Due to antimicrobial activity of some EOs, a different number of rows was used for biofilm data at 1.00% *v*/*v* (Tables SM5 and SM6). A further biofilm dataset (Table SM9, column C) was compiled by filling biofilm modulation data at 1.00% *v*/*v* with the correspondig values from data obtained at 0.05% *v*/*v* (Biofilm 1.00% *v*/*v* Corr). To evaluate the under developing ML model ability in discriminating either biofilm inhibiting or biofilm stimulating EOs, the biological data were binarized (partition into two classes) using different percentages of biofilm production threshold values (Table SM9). For all the used strains, threshold values of 40% (strong biofilm inhibition), 80% (moderate biofilm inhibition) and 120% (biofilm stimulation) were used. Trials to use 100% or median values of the biofilm production were also performed (Tables SM6 and SM7). The antimicrobial dataset was straightforwardly divided into active and inactive classes (Table SM10).

#### 2.5.2. Classification Models

To avoid too much unbalanced datasets, the modeling was restricted to binarized data showing at maximum a ratio of 10%:90% (or 90%:10%) data distribution. Therefore, considering the data reported as listed in Tables SM9 and SM10, among the 80 possible combinations (five strains by five thresholds [#: 1–25] by two biofilm data [A, B and C ] plus five antimicrobial [D]), seven of them were not considered, as the number of actives or inactives was not sufficient. Classification modeling was carried out with six different ML algorithms (RF, GB, SV, LR, DT and KNN) using the introduced datasets (Tables SM9 and SM10). Classification models were built with a number of latent variables corresponding to 85% of the whole chemical components variance extracted by PCA. Hyperparameter optimization was carried out with a wide range of settings (Table SM11), leading from hundreds of thousands to billions of combinations. Therefore, to speedup the optimization, the random search was used. Random search hyperparamenters’ optimization was proved, having a probability of 95% of finding a combination of parameters within the 5% optima with only 60 iterations, while reducing the probability to bog down in local optima [26]. Accordingly, herein 3000 random combination were used at four different nlevels (see Material and Methods) and, as a last step, the models from random search were refined by a grid search inspecting numerical hyperparameters in a range of ± 10. Thus, the initial random search hyperparameters’ tuning of 73 combinations led to 1752 (73 by six ML algorithms by four nlevels) classification models that were pruned on the basis of MCC and AUC cutoff values set to 0.4 and 0.5 [27,28,29,30], respectively (Tables SM12–SM18). As a result, the six ML algorithms led to define 104 statistically acceptable models (not shown). As different ML algorithms led to comparable models on the same dataset, and to avoid any redundancy, only those characterized by MCC higher than 0.5 were analyzed (models ML1–ML27 listed in Table 2). To this, FIs were inspected to investigate the most important chemical components likely responsible for biofilm modulation and antimicrobial activity. Moreover, PDs were finally investigated to seek for the statistical responsibility for each model’s most important chemical components.

##### Chemical Components Importance and Partial Dependences

**Figure 6:**
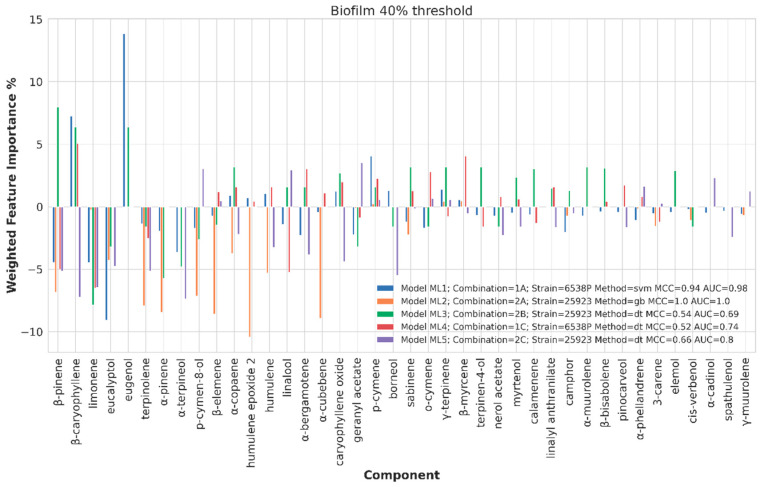
Weighted feature importance (WFI) plot for models obtained on the dataset binarized at 40% biofilm inhibition. Positive bars are associate with inhibition of biofilm production, whereas negative bars are associated with augmented biofilm production.

**Figure 7:**
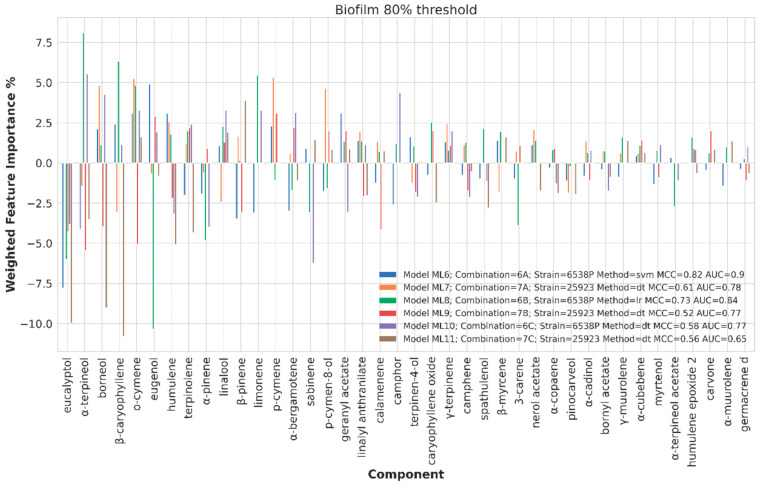
Weighted feature importance (WFI) plot for models obtained on the dataset binarized at 80% biofilm inhibition. Positive bars are associate with inhibition of biofilm production, whereas negative bars are associated with augmented biofilm production.

**Figure 8:**
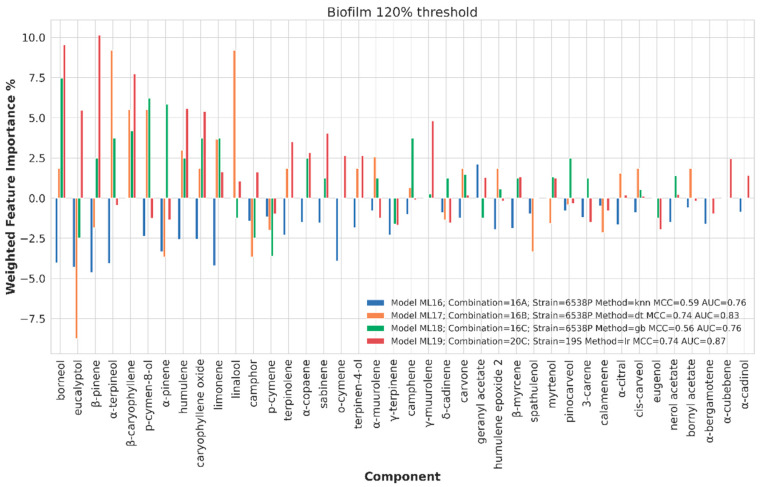
Weighted feature importance (WFI) plot for models obtained on the dataset binarized at 120% biofilm inhibition. Positive bars are associate with inhibition of biofilm production, whereas negative bars are associated with augmented biofilm production.

**Figure 9:**
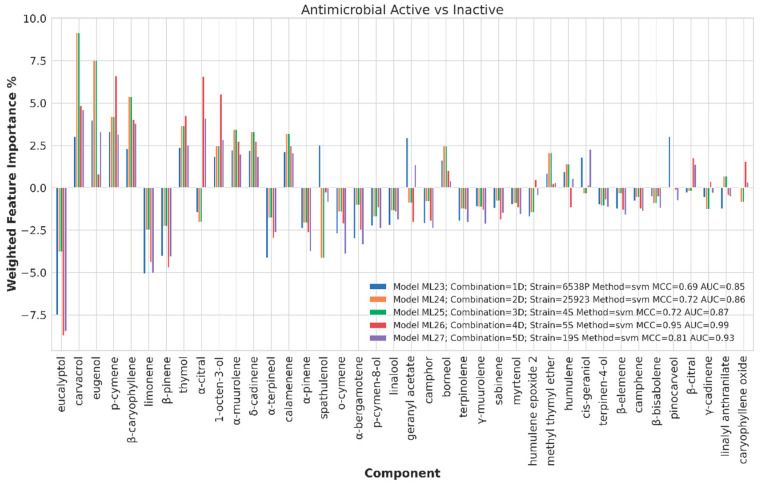
Weighted feature importance (WFI) plot for models obtained on active and inactive binarized dataset antimicrobial data. Positive bars are associate with antimicrobial activity, whereas negative bars indicate components with potential antisynergistic effect on *S. aureus* spp.

Chemical component importance was evaluated through FIs and PDs. Each FI indicates a sort of absolute correlation coefficient for each of the chemical components, while the associated PD gives its negative, positive or neutral influence. Therefore, PDs positive or negative trends were investigated by means of a spearman correlation (SP) coefficient, which is known to range from −1 to 1. The SP values were used to positively or negatively weight the corresponding FI values to obtain positive or negative weighted FIs (WFIs). WFIs were inspected by means of bar plots in a straightforward interpretation. For sake of clarity and redundancy avoidance, the analysis was focused on the top 30 FI values (Figure 6, Figure 7, Figure 8 and Figure 9). To avoid any recurrence and to reduce text length, only the detailed results for the 40%, 80% and 120% biofilm thresholds and for the antimicrobial data are reported. The overall associated effects for the chemical components are summarized in Table 3.

##### Chemical Components Importance and Partial Dependences at 40% Biofilm Production Threshold Value

At 40% biofilm production threshold value, acceptable MCC and AUC values were obtained for 6538P and 25293 *S. aureus* strains (ML1, ML2, ML3, ML4 and ML5, Table 2 and Figure 6). In general, an overall similar trend was observed for all the compiled dataset. In particular, β-caryophyllene (partially), eugenol and β-pinene (partially) components, at different tested concentrations, and listed in the top 30 most frequent EOS’ components with percentage of presence of 64%, 11% and 49%, respectively (Figure 5 and Table SM8), had a positive influence on the EO strong biofilm inhibition potency; among which eugenol showed the highest WFI value (about 14). On the contrary, eucalyptol, α-pinene, p-cymen-8-ol, terpinolene, humulene, β-elemene, humulene epoxide 2, α-cubebene, limonene, linalool, β-caryophyllene, α-terpineol, borneol and β-pinene (partially for 6538P), listed in the top 60 most frequent compounds (Table SM8), showed to have a variable negative impact on biofilm inhibition (Figure 6). Among the latter, eucalyptol, humulene epoxide 2 and α-cubebene displayed the lowest FWI values.

##### Chemical Components Importance and Partial Dependences at 80% Biofilm Production Threshold Value

At 80% biofilm production threshold value, ML models were obtained for the 6538P and 23923 strains (ML6, ML7, ML8, ML9 and ML10, Table 2). For an overall moderate biofilm inhibition, the most important constituent was indicated to be α-terpineol, present in 28 EOs (46%) with a WFI value of about 8 (Table SM8 and Figure 7). Other biofilm negatively modulating compounds, as indicated by the ML model, were o-cymene (partially), p-cymene, limonene and β-caryophyllene (partially).

On the contrary, eucalyptol, eugenol, α-terpineol, o-cymene (partially), sabinene, humulene, β-caryophyllene (partially) and borneol all has a negative influence on biofilm inhibition potency; eucalyptol being the most important in negatively influencing the anti-biofilm activity for both 6538P and 25923 strains.

##### Chemical Components Importance and Partial Dependences at 120% Biofilm Production Threshold Value

At threshold percentage of 120%, acceptable models (ML16, ML17, ML18 and ML19, Table 2) gave some hints on the chemical components mainly responsible for increased biofilm formation for 6538P and 19S strains. In particular, eucalyptol (in 33 EOs, Table SM8) seems to positively correlate with strong biofilm stimulation for both 6538P and 25923 reference strains (Figure 8 and Table 3). On the other hand, and in agreement with the previous analysis at 40% and 80% biofilm production threshold values, some compounds such as β-caryophyllene, α-terpineol, linalool, p-cymen-8-ol, borneol, α-pinene, β-pinene, eucalyptol (19S clinical strain), humulene and β-caryophyllene oxides are correlated with a non-stimulating biofilm producing effect. Furthermore, this model highly evidenced some controversy in the role of eucalyptol, which is correlated with promoting biofilm production for 6538P and is shown as a biofilm reducer from model ML19, which was developed arbitrarily with non-homogenous data.

##### Chemical Components Importance and Partial Dependences for Antimicrobial Activity

Differently from biofilm related datasets, ML models with very high statistical coefficients were built for all strains (Table SM17). Inspection of the representative models (ML28, ML29, ML30, ML31 and ML32, Table SM18) revealed the WFIs to share the same profile for almost all chemical components. Specifically, positive WFIs were calculated for eugenol, carvacrol, β-caryophyllene, p-cymene, 1-octen-3-ol and α-citral. Contrarily, strong negative WFIs were observed for eucalyptol, limonene and β-pinene (Figure 9), along with less important components such as α-terpineol, α-pinene and o-cymene.

## 3. Discussion

Bacterial growth in sessile phenotype (biofilm) plays a pivotal role in the chronicization of many infections, including lung infections, as in CF patients; it represents a form of strong phenotypical resistance to the host immune defenses and antibacterial drugs. The identification of new compounds able to inhibit biofilm growth could lead to remove a primary cause of the persistence of infections.

Recently [19] the antibacterial activity exerted by some selected EOs was demonstrated, from a list of 61, on the planktonic forms of *S. aureus* and *P. aeruginosa* strains isolated from CF patients. 

Here it is reported the investigation on the potential antibiofilm activity of the same EOs against a selected group of clinical isolates and reference *S. aureus* strains.

The results above reported showed the capability of two out of the 61 tested EOs (EO45 and EO58), at 1% concentration, to strongly reduce biofilm growth below 40% in all tested *S. aureus* strains, while only one essential oil (EO47) was effective in reducing biofilm growth below 50%. All the other EOs showed an extreme variability in biofilm modulation (positively and negatively) on the *S. aureus* strains. A different scenario was observed at 0.05% concentration: Almost all EOs lost their antibiofilm activity and some EOs stimulated biofilm growth. The equilibrium between formation and disruption of biofilm is subtle, being driven by a wide array of intracellular and extracellular factors. Therefore, it is not surprising that the same EO, a mixture of chemical compounds that may act synergistically or anti-synergistically, may perform as a biofilm growth inhibitor or activator, depending on the testing concentration.

The chemical analysis of the tested EOs coupled with ML modeling indicated that, among the 239 constituents, those related to strong biofilm growth inhibition below 40%, compared to untreated samples, were mainly eugenol, β-caryophyllene and partially β-pinene, while eucalyptol, α-pinene, p-cymen-8-ol, terpinolene, humulene, β-elemene, humulene epoxide 2, α-cubebene, limonene, linalool, α-terpineol and borneol were related to a non-antibiofilm growth role. These finding are in agreement with several recent reports. Purkait et al. [31] demonstrated the ability of eugenol and β-caryophyllene, alone or in combination, to reduce biofilm of *Listeria monocytogenes* and *Salmonella typhimurium*. Eugenol was demonstrated to show 17–86%, 24–69%, 30–91%, 9–94% and 4–89% reduction in biofilm biomass of *S. aureus* ATCC 25923 and several MRSA strains (FSA3, FSA11, FSA13 and FSA32), respectively [32]. Effect on biofilm of eugenol was studied in vitro using microtiter plate assay and in vivo on an otitis media-rat model, respectively. Sub-inhibitory concentration of eugenol significantly inhibited biofilms growth of MRSA and MSSA in vitro in a concentration-dependent manner; it decreased the expression of biofilm- and enterotoxin-related genes. Eugenol showed a synergistic effect with carvacrol on the eradication of pre-established biofilms [33]. Interestingly, β-caryophyllene and eugenol were also indicated by ML among those mainly involved in the antibacterial activity on the planktonic phenotype of the same bacterial strains [19]. Furthermore, ML designated the following antibacterial components as important: carvacrol, 1-octen-3-ol, α-citral and p-cymene. Scientific articles published in the last years have reported about either antibiofilm or antibacterial potencies of EO components. In particular, the inhibition of bacterial growth and biofilm production by β-caryophyllene on *Streptococcus mutans* has been recently reported [34]. Herein, carvacrol was predicted to be important for broad antibacterial activity on *S. aureus* 6538P; a report indeed indicated its experimental efficacy in both inhibition of biofilm production and antibacterial activity against *Salmonella enterica* serotype *Typhimurium* (ATCC 14028) [35]. A controversial profile was observed for limonene, predicted by ML to have a pro-biofilm effect by models developed at 40% threshold, an anti-biofilm role at 80% threshold and associated to an anti-synergistic effect on the antibacterial potency. Nevertheless, this unusual behavior was already reported in an investigation on 90 EOs against four *Staphylococcus* species (*S. aureus* 6538P, *S. aureus* 25923, *S. epidermidis* RP62A and *S. epidermidis* O-47) [18]. A behavior similar to that of limonene was also predicted for o-cymene and β-pinene. Several literature reports confirm the role of limonene in modulating biofilm production in other bacteria, thus highlighting its EO’s localized importance. Limonene showed a concentration-dependent reduction in the biofilm formation of *Streptococcus pyogenes* (SF370), with minimal biofilm inhibitory concentration (MBIC) of 400 µg mL^−1^. Limonene was found to possess about 75–95% antibiofilm activity against all the tested pathogens (ATCC 6249) [36]. Regarding the other components indicated important for the antimicrobial activity, such as 1-octen-3-ol and citral, reports clearly indicated some interesting activity against a series of bacteria, including *S. aureus* [37]. On the other hand, although negative data are difficult reported, herein was stressed by the ML model that eucalyptol (1,8-cineol) was indicated to likely have a detrimental effect on both biofilm production inhibition and antibacterial potency (Table 3). From a survey it was found indeed that eucalyptol was effectively reported to have scarce potency either on biofilm inhibition or as an antibacterial agent [38].

Planktonic bacteria continuously detach from a biofilm and spread out; as a consequence the whole bacterial population generated by a biofilm includes both planktonic and sessile cells that display different biological behaviors in metabolism, growth rate, resistance (both to host immune response and to antibacterials), etc. [39]. An ideal anti-infective strategy should aim both at preventing/reducing biofilm growth and at the killing of planktonic bacteria. Such a combination would likely restore the full efficacy of the host immune response. In this regards, an EO including components exerting both/either antibiofilm and/or antimicrobial activity could represent an effective tool to lower bacterial virulence and enhance antibacterial drug’s efficacy [40]. It could be speculated that the antibiofilm components might reduce bacterial aggregation, while antimicrobial ones would kill isolated bacteria.

In this report, the antibiofilm activity was assessed with the Christensen method and was confirmed by SEM investigations. The modifications of biofilm structure observed at SEM after treatment with EO45 and EO58 were similar. The chemical compositions of EO45 and EO58 were both qualitatively and quantitatively different, thus suggesting that different compounds might interfere with biofilm production. In this regard, and in agreement with ML suggestions, it could be therefore guessed that EOs’ antibiofilm activity is likely due to multiple mechanisms of action, in agreement with the multifactorial regulation of biofilm phenotype.

The reported data suggest that antibiofilm activity obtained at 1.00% concentration can be lost or even overturned at lower concentrations. At first glance, and according to the obtained experimental data, antibiofilm active EOs should be used only in modalities that do not reduce their concentration, as in diluted form they could induce biofilm growth. Therefore, their use should be limited to sanitary settings (as disinfectants of tools and surfaces) and for the topical treatment of human body surfaces. On the other hand, ML-based analysis suggested that some EO components could be of interest to reduce biofilm production and to increase antimicrobial potency. In this, mixtures of essential oils might represent a workaround. As greatly supported by literature, ML investigation and further experimental data could elucidate if combinations of carefully selected EOs could create synergies, allowing concentration reduction for an effective biofilm inhibition.

## 4. Materials and Methods 

### 4.1. Ethics Approval and Informed Consent

The approval for this research was granted by the Ethics Committee of Children’s Hospital and Institute Research Bambino Gesù in Rome, Italy (No 1437_OPBG_2017 of July 2017), and it was performed according to the principles of the Helsinki Declaration. Informed consent was obtained from all individual participants and all parents/legal guardians of the patients included in the study.

### 4.2. S. aureus Clinical Isolates from CF Patients Used for the Biofilm Production Assays

In this investigation, 3 representative *S. aureus* strains isolated from CF patients were used, selected from a list of 20 by means of unsupervised ML clusterization, as recently described [19]. Briefly, patients were treated according to current standards of care [41], with at least four microbiological controls per year. Informed consent was obtained from all subjects aged 18 years and older and from parents for all underage. According to the approved guidelines, microbiological cultures were performed using appropriate selective media and manual or automatic systems (API20NE, Vitek2, MALDI-TOF mass spectrometry); isolates were identified by 16S rRNA sequencing. The *S. aureus* strains were selected from a local collection containing about 10.000 CF bacterial isolates. The selected strains have different phenotypic and biochemical characteristics, in order to represent the complexity of the pulmonary microbial population of CF patients treated at the OPBG center. Additional data are reported in Supplementary Materials (Tables and Figures labeled as SM#). The 3 selected S. aureus strains were clustered on the basis of 13 qualitative descriptors (Table SM1). The phenotypic and genotypic characteristics of 3 representative *S. aureus* strains used in this work are summarized in Table SM2. As often reported [42,43], *S. aureus* ATCC 6538P (6538P) and *S. aureus* ATCC 25923 (25923) were also included in the study, used as reference strains for either antimicrobial or biofilm formation evaluation.

### 4.3. Biofilm Production Assay in Presence of EO

The quantification of in vitro biofilm production was based on microtiter plate biofilm assay (MTP). The wells of a sterile 96-well polystyrene flat-base plate were filled with medium containing a dilution of the bacterial culture in exponential growth phase in presence and absence of each EO as previously reported [18]. Briefly, the wells of a sterile 96-well flat-bottomed polystyrene plate were filled with 100 µL of the appropriate medium. 1/100 dilution of overnight bacterial cultures was added into each well (about 0.5 OD 600 nm). As control, the first row contained the untreated bacterial cells in Brain Hearth Infusion broth (BHI, Oxoid, Basingstoke, UK). In the second row the same culture medium was added with the addition of each EO at an appropriate concentration. The plates were aerobically incubated for 18 h at 37 °C. After the incubation, planktonic cells were gently removed; each well was washed three times with double-distilled water and patted dry with a piece of paper towel in an inverted position. For the quantification of biofilm formation, each well was stained with 0.1% crystal violet and incubated for 15 min at room temperature, rinsed twice with double-distilled water, and thoroughly dried. The remaining dye attached to the adherent cells was solubilized with 20% (*v*/*v*) glacial acetic acid and 80% (*v*/*v*) ethanol. After 30 min of incubation at room temperature, the total biomass of biofilm in each well was spectrophotometrically quantified at 590 nm. Each data point is composed of 4 independent experiments, each performed at least in 3 replicates.

For SEM analysis bacteria were grown as reported below; briefly, 1/100 dilution of overnight bacterial cultures was transferred in tubes containing SEM stubs (aluminum, 12.5 mm diameter, 6 mm pin) and incubated for 18 h at 37 °C in static conditions to assess biofilm production, in BHI in presence and in absence of EO. After the growth, SEM stubs were washed in 0.1 M phosphate buffer pH7.4 (PB) and fixed in glutaraldehyde 2.5% in 0.1 M PB buffer.

### 4.4. SEM Protocols

Samples of *S. aureus* 4S, *S. aureus* 5S and *S. aureus* 25923 grown on aluminum disks were processed as reported in Table SM3 with OsO_4_-RR-TA-IL protocol. 

As previously reported [44], the OsO4-RR-TA-IL protocol avoids dehydration, drying and sputter coating [45,46,47], allowing high-resolution and high-magnification imaging of biofilm three-dimensional structure without artifacts formation. In addition, it is a fast procedure, with low sample loss. The use of RR and TA, implemented by IL, lends the sample resistant under high vacuum and high voltages (15–20 kV) conditions.

### 4.5. Statistical Analysis of Biological Evaluation

Data reported were statistically validated using Student’s t-test comparing mean absorbance of treated and untreated samples. The significance of differences between mean absorbance values was calculated using a two-tailed Student’s *t*-test. A *p* value of <0.05 was considered significant.

### 4.6. Essential Oil Chemical Composition Analysis

EOs listed in Table SM4 were purchased from Farmalabor srl (Assago, Italy) and analyzed by gas chromatograph and mass spectrometer (GC-MS) to characterize their chemical composition. A Turbomass Clarus 500 GC-MS/GC-FID from Perkin Elmer instruments (Waltham, MA, USA), equipped with a Stabilwax fused-silica capillary column (Restek, Bellefonte, PA, USA) (60 m × 0.25 mm, 0.25 μm film thickness), was used to perform the chemical analyses. The operating conditions used were as follows: GC oven temperature was set at 40 °C for 5 min and programmed to 220 °C at a rate of 6 °C/min, and kept constant at 220 °C for 20 min. Helium was used as carrier gas (1.0 mL/min). Mass range was from 40 to 450 m/z using electron-impact at 70 eV mode. A total of 1 μL of each essential oil was diluted in 1 mL of methanol and 1 μL of the solution was injected into the GC injector at the temperature of 280 °C. Relative percentages for quantification of the components were calculated by electronic integration of the GC-FID peak areas. The identification of the constituents was achieved by comparing the obtained mass spectra for each component with those reported in mass spectra Nist 02 and Wiley libraries. Linear retention indices (LRIs) of each compound were also calculated using a mixture of aliphatic hydrocarbons (C8–C30, Ultrasci Bologna, Italy) injected directly into GC injector at the same operative conditions reported above. All analyses were repeated twice.

### 4.7. Machine Learning Binary Classification

Similarly as reported [18], all calculations were performed using the Python programming language (version 3.7, https://www.python.org/) by executing in-house code in the Jupyter Notebook platform. The biological data and essential oil chemical composition were imported and loaded into a Python Pandas dataframe and pre-processed to the final datasets to develop the classification models. Machine learning algorithms used in this study were implemented using the Scikit-learn library (sklearn) [48]. Unsupervised dimensionality reduction was performed with principal component analysis (PCA) [49] to extract 85% of explained variance. Cross-validation (CV) was used to evaluate the robustness of the final models as well as during the hyperparameters’ tuning. Different cut-off values were used to obtain the optimized hyperparameters classification models for each strain. A first hyperparameters selection was achieved through 3000 randomized runs from all possible considered combinations [50]. Furthermore, variables which take only a few values (nlevels) and, in addition, have ill distribution of the objects in these levels were pruned. Nlevel variables are dangerous as they force the under training model to fit most of the variance of a few objects with a high leverage, thus leading to spurious and misleading results. Column pruning was applied up to 4 unique levels. A final optimization was completed through a systematic variation (grid search) of the numerical random selected hyperparameters varying the values in a range of ± 10 (Table SM11). To develop the models different linear and non-linear ML classification algorithms were used: random forest (RF), gradient bosting (GB), support vector (SV), logistic regression (LR), decision tree (DT), and *k* nearest neighbors (KNN) as implemented in sklearn. The binary classification models were numerically and graphically evaluated by accuracy (ACC), F1 score, Matthews correlation coefficient (MCC), receiver operating characteristic (ROC) area under the curve (AUC). The importance of EOs chemical components was individually evaluated through the “feature importance” (FI) and partial dependence (PD) [51] as implemented in the Skater python library [52,53]. Internal models’ validation was carried out by leave-some-out CV using 5 groups using the stratified K-fold method while monitoring the average value of MCC obtained from 50 random CV iterations [17,54]. Final models were selected based on both MCC and ROC AUC values.

## Figures and Tables

**Table 1:** Chemical composition (%) of EO45 and EO58 obtained by GC-MS.

N°	Chemical Component ^1^	LRI ^2^	LRI ^3^	Peak Area (%)	
				EO45	EO58
1	α-pinene	1031	1035	6.9	1.0
2	β-pinene	1100	1105	6.3	3.8
3	sabinene	1103	1107	7.2	0.6
4	3-carene	1142	1146	5.9	-
5	limonene	1211	1210	11.1	8.8
6	β-ocimene	1242	1239	-	0.1
7	γ-terpinene	1251	1248	-	0.1
8	p-cymene	1290	1287	1.1	-
9	δ-elemene	1466	1465	2.7	-
10	α-cubebene	1482	1481	-	0.5
11	α-copaene	1485	1487	5.4	-
12	α-gurjunene	1530	1527	-	0.4
13	linalool	1533	1536	0.6	-
14	β-cubebene	1546	1541	0.7	-
15	α-bergamotene	1568	1566	0.1	-
16	β-elemene	1600	1598	0.7	-
17	cyclohexanone, 2-(1-methylethylidene)-	1611	*	-	1.0
18	terpinen-4-ol	1628	1630	0.4	0.7
19	myrtenal	1634	1632	-	0.3
20	β-caryophyllene	1638	1634	33.6	7.3
21	pulegone	1670	1665	-	59.8
22	γ-muurolene	1674	1676	0.2	-
23	α-muurolene	1692	1690	0.4	0.2
24	humulene	1694	1693	2.4	1.1
25	germacrene D	1728	1726	-	5.2
26	β-bisabolene	1733	1733	1.9	-
27	β-eudesmene	1748	1750	0.5	-
28	γ-cadinene	1751	1753	-	0.2
29	δ-cadinene	1760	1758	0.9	-
30	calamenene	1832	1827	0.3	0.7
31	jasmone	1952	1947	-	0.6
32	caryophyllene oxide	1963	1960	9.7	0.2
33	humulene epoxide 2	2038	2040	0.5	-
34	cubenol	2070	2074	-	0.2
35	spathulenol	2133	2136	0.4	-
36	τ-muurolol	2172	2178	-	0.1
37	thymol	2184	2189	-	1.2
38	cinerolon	2190	*	-	4.6
39	α-cadinol	2220	2218	-	0.2
40	epi-bicyclosesquiphellandrene	2230	*	-	0.9
	Total (%)			99.9	99.8

N°: The compound identification number; ^1^ the components are reported according their elution order on column; ^2^ linear retention indices measured on polar column; ^3^ linear retention indices from literature; * LRI not available for polar column; dash (-): traces < 0.1%.

**Table 2:** List of machine learning (ML) models selected by MCC and ROC AUC values. Random search selected best hyperparameters are also listed.

ML Id ^a^	Comb ^b^	Strain ^c^	ML Alg ^d^	Nlevel ^e^	MCC ^f^	AUC ^g^	Hyperparameters ^h^
ML1	1A	6538P	svm	4	0.94	0.98	P ^i^: True; k ^j^: rbf; Cw ^k^: {0: 1.0; 1: 3.0}; C ^l^: 1
ML2	2A	25923	gb	1	1	1	ne ^l^: 188; msl ^m^: 18; md ^n^: 33
ML3	2B	25923	dt	4	0.54	0.69	S ^v^: best; mss ^w^: 6; msl: 6; Mf ^x^: None; md: 1; cr ^y^: gini; cw: {0: 1.4; 1: 1.0}
ML4	1C	6538P	dt	1	0.52	0.74	cw: {0: 1.0; 1: 1.4}; cr: gini; md: 9; mf: None; msl: 5; mss: 4; s: best
ML5	2C	25923	dt	2	0.66	0.8	s: best; mss: 11; msl: 6; mf: None; md: 16; cr: entropy; cw: {0: 1.2; 1: 1.0}
ML6	6A	6538P	svm	1	0.82	0.9	p: True; k: rbf; cw: {0: 1.0; 1: 1.3}; C: 41
ML7	7A	25923	dt	3	0.61	0.78	s: best; mss: 15; msl: 3; mf: None; md: 20; cr: gini; cw: {0: 2.5; 1: 1.0}
ML8	6B	6538P	lr	1	0.73	0.84	Sl ^z^: newton-cg; pen ^aa^: l2; mi^bb^: 10000; cw: {0: 1.4; 1: 1.0}; C: 11
ML9	7B	25923	dt	3	0.52	0.77	s: best; mss: 6; msl: 1; mf: None; md: 6; cr: gini; cw: {0: 2.5; 1: 1.0}
ML10	6C	6538P	dt	2	0.58	0.77	s: best; mss: 11; msl: 11; mf: None; md: 11; cr: entropy; cw: 0: 1.5; 1: 1.0
ML11	7C	25923	dt	4	0.56	0.65	s: best; mss: 11; msl: 11; mf: None; md: 11; cr: entropy; cw: 0: 1.5; 1: 1.0
ML12	11A	6538P	svm	1	0.64	0.76	p: True; k: rbf; cw: 0: 1.0; 1: 2.0; C: 4
ML13	14A	5S	dt	4	0.68	0.74	cw: 0: 1.3; 1: 1.0; cr: gini; md: 14; mf: None; msl: 4; mss: 19; s: best
ML14	11B	6538P	gb	1	0.57	0.68	ne: 151; msl: 11; md: 61
ML15	15B	19S	dt	1	0.61	0.8	s: best; mss: 11; msl: 1; mf: None; md: 16; cr: entropy; cw: 0: 1.0; 1: 1.5
ML16	16A	6538P	knn	1	0.59	0.76	w: distance; p: 2; nn: 4; mp: None; m: manhattan; ls: 3; a: kd_tree
ML17	16B	6538P	dt	1	0.74	0.83	s: best; mss: 6; msl: 3; mf: None; md: 6; cr: entropy; cw: 0: 3.0; 1: 1.0
ML18	16C	6538P	gb	1	0.56	0.76	ne: 181; msl: 1; md: 31
ML19	20C	19S	lr	1	0.74	0.87	sl: saga; pen: l1; mi: 10000; cw: 0: 3.0; 1: 1.0; C: 51
ML20	23A	4S	gb	3	0.55	0.73	ne: 4; msl: 13; md: 72
ML21	22B	25923	dt	3	0.51	0.61	s: best; mss: 2; msl: 19; mf: None; md: 1; cr: gini; cw: 0: 1.0; 1: 1.2
ML22	22C	25923	dt	4	0.62	0.83	cw: 0: 1.0; 1: 1.0; cr: gini; md: 14; mf: None; msl: 10; mss: 4; s: best
ML23	1D	6538P	svm	3	0.69	0.85	p: True; k: rbf; cw: 0: 1.0; 1: 3.0; C: 1
ML24	2D	25923	svm	2	0.72	0.86	p: True; k: rbf; cw: 0: 1.1; 1: 1.0; C: 11
ML25	3D	4S	svm	2	0.72	0.87	p: True; k: rbf; cw: 0: 1.1; 1: 1.0; C: 11
ML26	4D	5S	svm	2	0.95	0.99	p: True; k: linear; cw: 0: 1.0; 1: 1.3; C: 1
ML27	5D	19S	svm	2	0.81	0.93	p: True; k: linear; cw: 0: 1.0; 1: 3.0; C: 1

^a^ Machine learning model id; ^b^ dataset combination as from Tables SM12–SM18; ^c^ *S aureus* strain code; ^d^ machine learning algorithm as defined in Material and Methods; ^e^ value to eliminate column with nlevel number of non-zero variables; ^f^ Matthew correlation coefficient; ^g^ ROC AUC values; ^h^ machine learning hyperparameters selected by random search optimization; ^i^, probability; ^j^, kernel; ^k^,class-weight; ^l^, C parameter; ^l^, number of estimators; ^m^, min_samples_leaf; ^n^, max_depth; ^o^, weights; ^p^, KNN p parameter; ^q^, number of neighbours; ^r^, metric parameter; ^s^, metric; ^t^, leaf size; ^u^, algorithm; ^v^, splitter; ^w^, min sample split; ^x^, max features; ^y^, criterion; ^z^, solver; ^aa^, penalty; ^bb^, max iters.

**Table 3:** Summary on the ML predicted essential oils’ (EO) single compounds influence on biofilm modulation and antibacterial activity. Compounds with an absolute value of FWI higher than 5 are listed.

	T ^a^	Anti-biofilm or Synergistic Antimicrobial ^b^	Spectrum ^c^	Pro-biofilm or Antisynergistic Antimicrobial ^d^	Spectrum
Biofilm modulation	40	eugenol	6538P, 25923	eucalyptol	6538P
		β-caryophyllene	6538P, 25923	β-pinene	6538P
		β-pinene	25923	α-pinene	25923
				p-cymen-8-ol	25923
				terpinolene	25923
				humulene	25923
				β-elemene	25923
				humulene epoxide 2	25923
				α-cubebene	25923
				limonene	25923, 6538P
				linalool	6538P
				α-terpineol	25923
				borneol	25923
	80	o-cymene	25923	eucalyptol	6538P, 25923
		p-cymene	25923	eugenol	6538P
		α-terpineol	6538P	α-terpineol	25923
		limonene	6538P	o-cymene	25923
		β-caryophyllene	6538P	sabinene	6538P
				humulene	25923
				β-caryophyllene	25923
				borneol	25923
	120	β-caryophyllene	6538P, 19S	eucalyptol	6538P
		α-terpineol	6538P		
		linalool	6538P		
		p-cymen-8-ol	6538P		
		borneol	6538P, 19S		
		α-pinene	6538P		
		β-pinene	19S		
		eucalyptol	19S		
		humulene	19S		
		caryophyllene oxide	19S		
Antibacterial activity	Active or Inactive	eugenol	25923, 4S	eucalyptol	6538P, 5S, 19S
		carvacrol	25923, 4S	limonene	6538P, 19S
		β-caryophyllene	25923, 4S	β-pinene	
		p-cymene	5S		
		1-octen-3-ol	5S		
		α-citral	5S		

^a^ Threshold value used to binarize the dataset response; ^b^ EOs’ components predicted mainly to act either to negatively modulate biofilm production or increase antibacterial potency; ^c^ indicates whether the component is important for all strains or a limited set; ^d,b^ EOs’ components predicted mainly to act either to positively modulate biofilm production or decrease antibacterial potency.

## Supplementary Materials

The following are available online at https://www.mdpi.com/1422-0067/21/23/9258/s1.
