# Supplementary material for: Essential Oils Biofilm Modulation Activity, Chemical and Machine Learning Analysis. Application on Staphylococcus aureus Isolates from Cystic Fibrosis Patients
Source: Int J Mol Sci. 2020 Dec 4;21(23):9258. doi: 10.3390/ijms21239258 (PMC7730550; doi:10.3390/ijms21239258)
Supplement: Supplementary file 1 [file ijms-21-09258-s001.pdf]

Essential oils biofilm modulation activity chemical and machine learning analysis. Application on *Staphylococcus aureus* isolates from cystic fibrosis patients.

Rosanna Papa[1,§], Stefania Garzoli[2,§], Gianluca Vrenna[3], Manuela Sabatino[4,5], Michela Relucenti[6], Orlando Donfrancesco[7], Ersilia Vita Fiscarelli[8], Marco Artini[9], Laura Selan[10,*] and Rino Ragno[11,12,*]

Rosanna Papa[1,§], Stefania Garzoli[2,§], Gianluca Vrenna[3], Manuela Sabatino[4,5], Michela Relucenti[6], Orlando Donfrancesco[7], Ersilia Vita Fiscarelli[8], Marco Artini[9], Laura Selan[10,*] and Rino Ragno[11,12,*]

[1] Department of Public Health and Infectious Diseases, Sapienza University, p.le Aldo Moro 5, 00185 Rome, Italy; rosanna.papa@uniroma1.it

[2] Department of Drug Chemistry and Technology, Sapienza University, p.le Aldo Moro 5, 00185 Rome, Italy; stefania.garzoli@uniroma1.it

[3] Department of Public Health and Infectious Diseases, Sapienza University, p.le Aldo Moro 5, 00185 Rome, Italy; gianluca.vrenna@uniroma1.it

[4] Department of Drug Chemistry and Technology, Sapienza University, p.le Aldo Moro 5, 00185 Rome, Italy; manuela.sabatino@uniroma1.it

[5] Rome Center for Molecular Design, Department of Drug Chemistry and Technology, Sapienza University, p.le Aldo Moro 5, 00185 Rome, Italy; manuela.sabatino@uniroma1.it

[6] Department of Anatomy, Histology, Forensic Medicine and Orthopaedics, Sapienza University of Rome, via Alfonso Borelli 50, Rome, 00161, Italy; michela.relucenti@uniroma1.it

[7] Department of Anatomy, Histology, Forensic Medicine and Orthopaedics, Sapienza University of Rome, via Alfonso Borelli 50, Rome, 00161, Italy; orlando.donfrancesco@uniroma1.it

[8] Paediatric and Laboratory Department. Children's Hospital and Institure Research Bambino Gesù, Italy; evita.fiscarelli@opbg.net

[9] Department of Public Health and Infectious Diseases, Sapienza University, p.le Aldo Moro 5, 00185 Rome, Italy; marco.artini@uniroma1.it

[10] Department of Public Health and Infectious Diseases, Sapienza University, p.le Aldo Moro 5, 00185 Rome, Italy; laura.selan@uniroma1.it

[11] Department of Drug Chemistry and Technology, Sapienza University, p.le Aldo Moro 5, 00185 Rome, Italy; rino.ragno@uniroma1.it

[12] Rome Center for Molecular Design, Department of Drug Chemistry and Technology, Sapienza University, p.le Aldo Moro 5, 00185 Rome, Italy; rino.ragno@uniroma1.it

Table SM1. Qualitative descriptors used for the unsupervised Machine Learning clusterization of *S. aureus* strains.

**Descriptor Name**	**Parameter**
Type of sample	Esp: sputum; AT: hypopharyngeal suction
Methicillin profile	MRSA e MSSA
Phenotype	Small colony variant, normal
Quinolones Sensitivity	Resistant or Susceptible
Trimethoprim/Sulfamethoxazole Sensitivity	Resistant or Susceptible
Erythromycin Sensitivity	Resistant or Susceptible
Clindamycin Sensitivity	Resistant or Susceptible
Linezolid Sensitivity	Resistant or Susceptible
Inducible Clindamycin resistance ability	Positive or negative
Fungal Co-infection	*Scedosporium prolificans*
P. aeruginosa co-infection	Positive or negative
cftr gene mutation	621+1G>T/R553X<br>F508del/F508del<br>L636P/P499A
Biofilm formation	Strong, moderate or weak

Table SM2. Phenotypical and genotypical characterization of 3 representative strains of *S. aureus.*

ID pt	ID	SAM	Date	Str	Ph	QUIN	B	ER	CLI	LIN	RCLI	CF	CPA	GENOTYPE
4	4S	AT	2/20/2009	MRSA	-	S	S	Nt	S	S	P			621+1G>T/R553X
5	5S	ESP	11/13/2009	MRSA	-	R	S	Nt	R	S	N	Sp		F508del/F508del
19	19S	ESP	6/15/2017	MSSA	-	S	S	R	R	S	P		X	L636P/P499A

The 3 *Staphylococcus aureus* clinical isolates and their characterization by several properties. **ID pt:** Patient Identification; **ID**: Strain Code; **SAM**: Sample; **Date**: Date of collection; **Str**: Strain; **Ph**: Phenotype; **QUIN**: Quinolones; **B**: Trimethoprim/Sulfamethoxazole; **ER**: Erythromycin; **CLI**: Clindamycin; **LIN**: Linezolid; **RCLI**: Inducible Clindamycin resistance; **CF**: Fungal Co-infection; **CPA**: *P. aeruginosa* co-infection; **GEN**: Pts Genotype; **Esp**: Sputum; **AT**: Hypopharyngeal Suction; **MRSA**: Methicillin Resistant *S. aureus*; **MSSA**: Methicillin Sensitive *S. aureus*;; **R**: Resistant; **S**: Susceptible; **N**: Negative; **Nt**: Non tested; Sp*: Scedosporium prolificans.*

Table SM3. SEM OsO_4-RR-TA-IL protocol and operating conditions for observation.

**Steps**	**OsO_4-RR-TA-IL Protocol**
**Fixation**	Glutharaldehyde 2,5% in PB 0.1 M pH 7.4 at least 48 h
**Washing**	10 min x2 times in PB 0.1 M pH 7.4
**Postfixation**	OsO_4 2%+RR 0.2% 1:1 solution, 1 h
**Washing**	10 min -2 times in H_20
**Impregnation**	Tannic Acid 1% in d H_20 30 min
**Washing**	10 min -2 times in H_20
**Dehydration**	None
**Drying**	None
**Pt Sputter coating**	Replaced by IL
**Operating conditions**	15-20 kV, high vacuum

Table SM4. ID and plant name of essential oils used in the investigation.

EO ID	EO Name	EO ID	EO Name
EO1	Chamomile Morocco	EO32	Birch
EO2	Sage Sclarea	EO33	Fennel
EO3	Salvia Officinalis	EO34	Cedar Fruit
EO4	Red Thyme	EO35	Lemon
EO5	Tea Tree Oil	EO36	Roman Chamomile
EO6	Melissa Oiio Essential	EO37	Savory
EO7	Pinus Mugo	EO38	Rosemary
EO8	Geranium Bourbon	EO39	Ceylon Cinnamon Peel
EO9	Oregano	EO40	Eucaliptus Globulus
EO10	Ylang Ylang	EO41	Sweet Orange
EO11	Coriander	EO42	Niaouly
EO12	Lavandula Angustifoglia	EO43	Artemisia
EO13	Myrtle	EO44	Cajeput
EO14	Garlic	EO45	Black Pepper
EO15	Cardamom	EO46	White Thyme
EO16	Mandarin	EO47	Marjoram
EO17	Hyssop	EO48	Cloves
EO18	Grapefruit	EO49	Cypress
EO19	Cymbopogon	EO50	Nutmeg Natural
EO20	Pinus Sibirica	EO51	Peppermint
EO21	Camphor	EO52	Verbena officinalis
EO22	Cadè	EO53	Basil
EO23	Cedar Leaves	EO54	Cymbopogon martinii
EO24	Ginger	EO55	Laurel
EO25	Cumin	EO56	Anise
EO26	Patchouli	EO57	Incense
EO27	Bitter Orange	EO58	Mentha Suaveolens
EO28	Eucalyptus	EO59	Coridotthymus Capitatus
EO29	Pinus Silvester	EO60	Thymus Vulgaris
EO30	Bergamot	EO61	Origanum Hirtum
EO31	Juniper		

Table SM5. Antimicrobial activity of EOs listed in Table S4**Errore. L'origine riferimento non è stata trovata.** on representative clinical strains and reference strains of *S. aureus* [19].

EOs ID	6538P	25923	4S	5S	19S
1	Antibacterial	Antibacterial	Antibacterial	Inactive	Inactive
2	Inactive	Inactive	Inactive	Inactive	Inactive
3	Inactive	Inactive	Inactive	Inactive	Inactive
4	Antibacterial	Antibacterial	Antibacterial	Antibacterial	Antibacterial
5	Inactive	Inactive	Inactive	Inactive	Inactive
6	Inactive	Antibacterial	Antibacterial	Antibacterial	Antibacterial
7	Inactive	Inactive	Inactive	Inactive	Inactive
8	Inactive	Inactive	Inactive	Inactive	Inactive
9	Antibacterial	Antibacterial	Antibacterial	Antibacterial	Antibacterial
10	Inactive	Inactive	Inactive	Inactive	Inactive
11	Inactive	Inactive	Inactive	Inactive	Inactive
12	Inactive	Inactive	Inactive	Inactive	Inactive
13	Inactive	Inactive	Inactive	Inactive	Inactive
14	Antibacterial	Antibacterial	Antibacterial	Antibacterial	Antibacterial
15	Inactive	Inactive	Inactive	Inactive	Inactive
16	Inactive	Inactive	Inactive	Inactive	Inactive
17	Inactive	Inactive	Inactive	Inactive	Inactive
18	Inactive	Inactive	Inactive	Inactive	Inactive
19	Inactive	Inactive	Inactive	Inactive	Inactive
20	Inactive	Inactive	Inactive	Inactive	Inactive
21	Inactive	Inactive	Inactive	Inactive	Inactive
22	Antibacterial	Antibacterial	Antibacterial	Antibacterial	Antibacterial
23	Inactive	Inactive	Inactive	Inactive	Inactive
24	Inactive	Inactive	Inactive	Inactive	Inactive
25	Inactive	Inactive	Inactive	Inactive	Inactive
26	Inactive	Inactive	Inactive	Inactive	Inactive
27	Inactive	Inactive	Inactive	Inactive	Inactive
28	Inactive	Inactive	Inactive	Inactive	Inactive
29	Inactive	Inactive	Inactive	Inactive	Inactive
30	Inactive	Inactive	Inactive	Inactive	Inactive
31	Inactive	Inactive	Inactive	Inactive	Inactive
32	Antibacterial	Antibacterial	Antibacterial	Antibacterial	Antibacterial
33	Inactive	Inactive	Inactive	Inactive	Inactive
34	Inactive	Inactive	Inactive	Inactive	Inactive
35	Inactive	Inactive	Inactive	Inactive	Inactive
36	Inactive	Inactive	Inactive	Inactive	Inactive
37	Antibacterial	Antibacterial	Antibacterial	Antibacterial	Antibacterial
38	Inactive	Inactive	Inactive	Inactive	Inactive
39	Antibacterial	Antibacterial	Antibacterial	Antibacterial	Antibacterial
40	Inactive	Inactive	Inactive	Inactive	Inactive
41	Inactive	Inactive	Inactive	Inactive	Inactive
42	Inactive	Inactive	Inactive	Inactive	Inactive
43	Inactive	Inactive	Inactive	Inactive	Inactive
44	Inactive	Inactive	Inactive	Inactive	Inactive
45	Inactive	Inactive	Inactive	Inactive	Inactive
46	Antibacterial	Antibacterial	Antibacterial	Antibacterial	Antibacterial
47	Inactive	Inactive	Inactive	Inactive	Inactive
48	Antibacterial	Antibacterial	Antibacterial	Antibacterial	Antibacterial
49	Inactive	Inactive	Inactive	Inactive	Inactive
50	Inactive	Inactive	Inactive	Inactive	Inactive
51	Inactive	Inactive	Inactive	Inactive	Inactive
52	Inactive	Inactive	Inactive	Antibacterial	Antibacterial
53	Inactive	Inactive	Inactive	Inactive	Inactive
54	Antibacterial	Inactive	Inactive	Inactive	Antibacterial
55	Inactive	Inactive	Inactive	Inactive	Inactive
56	Inactive	Inactive	Inactive	Inactive	Inactive
57	Inactive	Inactive	Inactive	Inactive	Inactive
58	Inactive	Inactive	Inactive	Inactive	Inactive
59	Antibacterial	Antibacterial	Antibacterial	Antibacterial	Antibacterial

60	Inactive	Inactive	Inactive	Antibacterial	Antibacterial
61	Antibacterial	Inactive	Inactive	Antibacterial	Inactive

Table SM6. Percentage of bacterial biofilm formation in the presence of each EO listed in Table S4 at concentration 1.00% v/v relative to untreated bacteria. Bacterial strains: *S. aureus* ATCC 6538P and *S. aureus* ATCC 25923, *S. aureus* 4S, *S. aureus* 5S and *S. aureus* 19S. Each data point is composed of 4 independent experiments, each performed with at least three replicates. The mean values are listed along with the calculated standard deviations (SD).

EOs ID	***S. aureus* strains**									
	**6538P**		**25923**		**4S**		**5S**		**19S**	
	Mean	SD	Mean	SD	Mean	SD	Mean	SD	Mean	SD
**1**	NA	NA	NA	NA	NA	NA	97.73	13.59	77.65	23.82
**2**	17.65	8.04	109.52	18.54	108.40	28.53	100.49	11.14	87.76	12.18
**3**	5.98	0.93	86.15	3.94	34.27	6.15	74.62	4.28	11.65	0.32
**4**	NA	NA	NA	NA	NA	NA	NA	NA	NA	NA
**5**	46.75	8.05	116.19	4.66	34.88	12.10	74.41	14.37	74.84	15.74
**6**	20.86	1.29	NA	NA	NA	NA	NA	NA	NA	NA
**7**	27.32	2.56	102.06	6.62	62.83	5.78	101.88	10.02	68.40	7.16
**8**	66.27	8.13	64.83	9.79	81.04	6.94	120.85	15.29	67.67	7.40
**9**	NA	NA	NA	NA	NA	NA	NA	NA	NA	NA
**10**	36.33	5.92	80.39	5.60	54.27	14.79	61.74	8.16	78.10	21.33
**11**	5.64	1.11	58.98	3.56	49.26	11.24	63.88	6.90	23.88	8.33
**12**	41.65	13.47	98.21	8.46	70.87	1.31	69.10	4.08	82.61	5.72
**13**	7.15	0.71	81.27	3.62	70.78	15.26	74.28	15.89	30.87	7.36
**14**	NA	NA	NA	NA	NA	NA	NA	NA	NA	NA
**15**	16.85	10.62	81.81	5.31	49.82	9.40	58.81	8.11	41.19	18.08
**16**	47.48	8.77	217.49	11.25	52.61	9.15	91.81	12.97	75.44	21.36
**17**	14.36	7.84	80.85	15.12	75.61	6.26	72.99	5.51	82.05	4.28
**18**	64.47	3.83	141.11	19.59	39.23	5.34	62.52	11.97	82.69	14.52
**19**	35.75	3.12	32.51	4.45	54.17	8.33	60.48	7.59	81.17	9.64
**20**	55.81	2.55	211.67	18.55	57.18	5.82	91.18	13.41	114.73	16.47
**21**	57.92	13.33	141.14	7.78	65.74	6.01	205.38	19.38	100.03	17.48
**22**	NA	NA	NA	NA	NA	NA	NA	NA	NA	NA
**23**	168.07	4.85	94.70	2.24	80.65	8.23	66.17	3.83	93.75	17.00
**24**	16.56	2.71	72.53	3.06	36.19	6.34	31.72	1.88	53.00	10.41
**25**	7.56	0.88	29.76	8.43	99.13	19.23	57.63	9.26	25.33	2.29
**26**	7.52	0.62	346.73	6.35	14.97	1.11	48.41	1.21	38.46	7.32
**27**	13.46	0.75	57.88	12.91	28.18	8.35	32.42	12.80	55.90	0.76
**28**	172.60	0.40	76.21	2.15	106.80	17.54	78.15	4.99	108.47	14.10
**29**	48.04	2.37	89.89	9.17	57.81	1.70	100.87	5.18	66.71	2.83
**30**	41.27	8.28	97.98	16.34	70.67	16.35	200.34	22.82	78.20	8.21
**31**	69.71	19.66	58.36	4.05	79.57	15.17	157.62	11.98	97.02	14.90
**32**	NA	NA	NA	NA	NA	NA	NA	NA	NA	NA
**33**	148.88	15.05	64.11	4.79	70.65	3.93	189.08	15.76	135.61	21.86
**34**	24.79	5.08	162.31	15.36	28.14	7.18	33.96	5.09	36.67	3.26
**35**	48.04	9.26	89.89	4.29	57.81	4.54	100.87	10.06	66.71	3.70
**36**	50.48	4.74	157.31	13.26	30.91	1.86	38.94	7.99	40.88	9.48
**37**	NA	NA	NA	NA	NA	NA	NA	NA	NA	NA
**38**	5.60	1.09	46.71	20.34	28.68	11.30	68.31	15.42	12.83	3.37
**39**	NA	NA	NA	NA	NA	NA	NA	NA	NA	NA
**40**	48.25	5.73	119.87	13.19	65.10	5.60	17.,39	10.08	70.58	4.20
**41**	30.60	8.35	61.90	9.02	89.66	15.45	36.25	10.47	119.29	12.75
**42**	55.30	10.99	80.86	2.09	89.41	9.31	143.41	5.50	74.34	6.19
**43**	30.45	6.28	56.99	5.90	108.71	14.92	75.51	2.46	96.23	14.76
**44**	45.77	14.40	50.08	16.13	64.64	17.02	60.51	4.58	96.96	16.93
**45**	4.72	0.69	36.24	15.21	34.85	15.73	32.23	13.35	17.07	5.52
**46**	NA	NA	NA	NA	NA	NA	NA	NA	NA	NA
**47**	10.08	1.37	46.59	14.20	40.78	19.99	38.46	15.12	18.04	3.72
**48**	NA	NA	NA	NA	NA	NA	NA	NA	NA	NA
**49**	267.37	3.58	31.48	12.65	79.44	13.07	101.06	5.93	143.09	9.16
**50**	84.44	12.16	53.16	9.38	90.01	13.94	64.02	21.78	64.63	7.54
**51**	21.14	12.27	49.12	6.98	90.36	18.14	62.49	8.98	63.16	5.22
**52**	16.21	8.11	59.86	7.09	55.13	2.49	NA	NA	NA	NA
**53**	32.05	6.30	36.17	2.71	61.13	3.99	42.67	2.42	69.56	7.91
**54**	NA	NA	27.97	1.38	47.85	8.09	25.21	0.96	NA	NA

**55**	145.12	17.00	33.92	3.39	58.11	5.39	71.55	9.00	93.09	21.61
**56**	127.30	11.50	65.23	2.99	90.94	10.39	75.24	14.78	123.07	17.95
**57**	7.65	1.61	65.02	4.20	57.74	7.35	92.57	15.58	82.20	5.83
**58**	4.46	0.69	22.83	12.78	25.10	7.09	38.38	13.46	13.51	1.38
**59**	NA	NA	NA	NA	NA	NA	NA	NA	NA	NA
**60**	6.59	0.76	61.09	4.39	52.16	3.77	NA	NA	NA	NA
**61**	NA	NA	38.42	2.13	83.56	14.98	NA	NA	121.45	9.14
**# Values**	48		49		49		47		47	48
**median**	131.5		79.23		96.95		119.34		99.94	131.5
**Median Corr**	31.13		65.02		65.1		74.93		82.05	31.13

Table SM7. Percentage of bacterial biofilm formation in the presence of each EO listed in Table S4 at concentration 0.05% v/v relative to untreated bacteria. Bacterial strains: *S. aureus* ATCC 6538P and *S. aureus* ATCC 25923, *S. aureus* 4S, *S. aureus* 5S and *S. aureus* 19S. Each data point is composed of 4 independent experiments, each performed with at least three replicates. The mean values are listed along with the calculated standard deviations (SD).

**EOs ID**	***S. aureus* strains**									
	**6538P**		**25923**		**4S**		**5S**		**19S**	
	Mean	SD	Mean	SD	Mean	SD	Mean	SD	Mean	SD
**1**	135.03	11.02	80.21	9.52	102.47	11.03	111.15	4.45	77.13	6.88
**2**	113.75	14.12	97.93	7.03	105.31	13.17	110.31	14.98	89.80	10.10
**3**	70.40	19.49	81.04	2.80	110.08	14.32	136.43	15.04	126.19	14.83
**4**	28.24	3.91	34.89	2.16	81.10	2.17	117.42	17.82	95.52	15.10
**5**	59.07	14.39	67.59	17.86	95.10	1.49	140.99	13.57	99.94	11.11
**6**	37.77	12.45	74.20	3.22	81.27	8.09	126.40	15.52	95.33	18.47
**7**	87.94	19.53	79.23	9.62	77.49	6.27	143.71	15.68	97.43	12.13
**8**	66.08	13.13	72.43	8.82	78.73	4.75	107.67	11.14	98.57	19.25
**9**	18.83	0.65	16.38	3.87	58.07	1.72	71.29	11.15	84.88	18.57
**10**	144.14	14.55	86.60	11.64	86.42	1.47	90.25	14.19	82.50	7.32
**11**	141.67	9.05	64.94	2.74	79.95	5.83	126.16	7.63	76.85	13.42
**12**	132.58	9.58	68.17	4.45	83.30	1.33	121.08	6.29	111.25	14.09
**13**	98.21	2.02	76.85	4.48	93.50	12.90	135.45	11.52	97.52	7.47
**14**	126.24	13.22	51.11	6.95	95.28	14.29	144.57	15.18	106.08	24.37
**15**	131.50	10.11	59.06	6.96	104.54	8.71	131.08	2.31	91.93	4.54
**16**	159.66	5.62	74.07	20.36	86.79	3.93	161.38	12.34	91.74	9.16
**17**	113.90	1.05	73.84	16.70	89.48	1.03	147.96	3.46	82.87	16.12
**18**	103.23	11.41	61.92	14.20	74.27	8.90	171.62	17.51	85.13	14.70
**19**	169.86	19.53	90.88	16.47	77.24	2.57	93.30	10.33	90.10	7.63
**20**	158.00	20.95	69.83	14.43	92.21	11.34	143.89	10.27	95.57	4.89
**21**	171.07	14.48	77.25	2.80	80.76	11.71	140.55	4.61	116.49	8.30
**22**	84.73	7.49	95.99	10.66	84.98	2.54	133.61	7.27	159.18	35.47
**23**	171.77	12.76	76.56	7.94	82.22	9.80	162.48	8.19	100.53	14.59
**24**	165.90	12.16	72.29	15.22	94.10	6.28	128.64	19.79	94.47	3.20
**25**	150.26	19.92	71.32	2.78	96.95	6.06	140.68	11.70	94.58	6.34
**26**	136.17	12.17	79.89	1.58	82.27	14.13	101.60	4.06	137.50	19.22
**27**	138.63	15.76	103.92	16.36	102.83	0.38	144.65	14.62	105.29	18.64
**28**	140.48	16.89	83.31	9.58	97.12	13.05	134.48	17.79	97.23	11.69
**29**	148.46	14.81	80.68	2.68	104.29	12.40	130.03	9.65	90.49	16.19
**30**	140.50	15.82	85.28	8.93	115.31	16.19	122.47	10.01	112.01	12.93
**31**	128.06	14.42	56.04	18.66	125.86	16.83	90.33	8.43	108.56	10.54
**32**	98.75	10.33	79.98	9.82	76.00	2.61	105.70	13.39	120.50	19.14
**33**	137.24	18.95	76.93	5.45	97.87	9.90	119.77	14.80	98.36	16.51
**34**	135.25	12.55	76.57	4.70	93.34	16.50	127.73	5.18	95.91	2.92
**35**	133.13	9.83	80.49	3.49	98.54	2.07	112.77	15.39	106.28	13.47
**36**	129.54	13.57	85.70	3.65	88.63	10.48	114.68	2.28	101.05	13.11
**37**	26.61	3.16	33.89	6.75	122.88	10.70	91.74	9.11	77.76	10.48
**38**	98.62	19.73	116.68	14.38	91.26	14.85	107.57	6.29	148.92	13.50
**39**	14.45	1.69	41.84	3.24	98.42	6.48	149.59	15.40	150.48	24.73
**40**	153.87	18.24	103.74	4.23	126.13	28.79	128.35	16.55	146.65	19.76
**41**	182.91	14.78	127.44	20.69	119.87	8.82	73.91	11.42	135.03	11.86

**42**	177.84	20.39	105.17	2.23	122.90	16.76	110.24	10.13	145.96	20.05
**43**	174.00	13.00	110.57	8.41	128.05	9.27	119.34	4.01	127.73	1.51
**44**	189.15	24.74	108.57	3.89	124.22	18.79	120.79	15.69	122.34	14.38
**45**	145.90	19.48	97.22	16.93	41.70	2.56	107.36	5.15	145.20	16.41
**46**	25.73	1.69	34.16	8.41	128.20	25.48	85.26	1.41	84.31	7.72
**47**	56.40	14.16	86.49	2.41	102.25	30.06	107.60	5.72	107.55	18.55
**48**	25.00	1.90	93.20	5.18	122.70	15.89	88.74	9.47	109.67	15.14
**49**	120.18	17.90	102.73	11.27	144.27	15.25	104.41	11.66	111.80	6.01
**50**	124.03	19.29	156.94	13.36	123.96	10.85	96.21	9.63	120.98	13.59
**51**	137.65	19.52	114.17	10.27	111.24	9.19	111.93	9.90	115.81	12.05
**52**	58.72	12.64	99.47	7.92	116.11	11.62	122.86	11.56	108.43	13.30
**53**	164.31	10.61	107.73	4.89	122.89	9.81	126.40	14.00	107.99	11.05
**54**	164.94	14.00	135.94	3.80	34.63	3.03	86.44	4.76	118.41	16.60
**55**	187.74	1.20	75.60	11.45	109.22	14.62	87.41	4.94	87.14	6.01
**56**	200.70	18.95	69.44	3.95	108.19	13.04	112.60	5.56	87.13	5.59
**57**	80.55	3.07	89.36	4.48	111.99	16.37	71.90	10.05	100.00	15.62
**58**	90.44	12.94	76.02	2.07	120.73	16.97	175.17	12.19	91.59	4.81
**59**	29.84	0.98	22.55	1.88	50.81	7.98	66.17	7.97	99.43	4.14
**60**	35.99	3.23	36.93	3.93	75.54	7.20	91.75	11.03	96.80	9.34
**61**	31.13	2.82	29.41	2.63	72.04	12.05	94.06	19.81	94.35	9.03
**Median**	131.5		79.23		96.95		119.34		99.94	131.5

Table SM8. Occurrences of the EOs' chemical components. Only the most frequent compounds are listed.

Component	#[a]	Component	#[a]	Component	#[a]
limonene	51	p-cymen-8-ol	10	linalyl anthranilate	6
α-pinene	47	terpinolene	9	calamenene	6
β-caryophyllene	39	α-citral	9	β-phellandrene	6
β-myrcene	36	β-bisabolene	9	3-carene	6
linalool	35	carvacrol	8	α-cadinol	6
eucalyptol	33	α-muurolene	8	spathulenol	6
β-pinene	30	γ-muurolene	8	germacrene d	6
o-cymene	29	carvone	8	α-phellandrene	5
α-terpineol	28	bornyl acetate	8	nerol acetate	5
caryophyllene oxide	27	eugenol	7	myrtenol	5
terpinen-4-ol	26	thymol	7	cis-β-farnesene	5
sabinene	22	α-bergamotene	7	γ-cadinene	5
γ-terpinene	21	geranyl acetate	7	estragole	5
borneol	16	camphor	7	cis-β-terpineol	5
humulene	16	pinocarveol	7	cis-verbenol	5
δ-cadinene	13	α-terpinene	7	elemol	4
camphene	13	α-terpineol acetate	7	α-cubebene	4
cis-geraniol	12	1-octen-3-ol	6	cis-carveol	4
p-cymene	11	β-elemene	6	β-citral	4
α-copaene	11	humulene epoxide 2	6	methyl thymyl ether	4

Table SM9. Description of the datasets used to develop the classification models for the biofilm data.

#[a]	Strain	Threshold[b]	A[a] Biofilm Modulation at 0.05 % v/v[c]		B Biofilm Modulation at 1.00 % v/v[d]		C Biofilm Modulation at 1.00 % v/v Corr[e]	
			**Negative**	**Positive**	**Negative**	**Positive**	**Negative**	**Positive**
**1**	6538P	40%	10	51	26	22	34	27
**2**	25923		7	54	9	40	14	47
**3**	4S		1	60	11	38	11	50
**4**	5S		0	61	9	38	9	52
**5**	19S		0	61	10	37	10	51
**6**	6538P	80%	15	46	41	7	49	12
**7**	25923		33	28	27	22	36	25
**8**	4S		12	49	37	12	40	21
**9**	5S		4	57	31	16	33	28
**10**	19S		3	58	28	19	29	32
**11**	6538P	100%	22	39	42	6	52	9
**12**	25923		49	12	38	11	50	11
**13**	4S		35	26	46	3	54	7
**14**	5S		15	46	35	12	42	19
**15**	19S		32	29	39	8	46	15
**16**	6538P	120%	25	36	42	6	52	9
**17**	25923		58	3	42	7	54	7
**18**	4S		49	12	49	0	58	3
**19**	5S		32	29	40	7	49	12
**20**	19S		48	13	43	4	54	7
**21**	6538P	median	31	30	24	24	31	30
**22**	25923		31	30	25	24	31	30
**23**	4S		31	30	25	24	31	30
**24**	5S		31	30	24	23	31	30
**25**	19S		31	30	24	23	31	30

a) dataset numbers and letters used for the dataset combination: 1A, 2A….1B, 2B….1C,2C…25C;
b) Threshold used to binarize data;
c) column indicating the number of samples showing biofilm growth modulation, negative or positive at the selected threshold at 0.05% v/v concentration;
d) column indicating the number of samples showing biofilm growth modulation, negative or positive at the selected threshold 1.00% v/v concentration;
e) column indicating the number of samples showing biofilm growth modulation, negative or positive at the selected threshold at 1.00% v/v concentration implemented with data at 0.05% v/v for missing values.

Table SM10. Description of the datasets used to develop the classification models for the antimicrobial data.

**#**[a]	**Strain**	**D**[b]	
		**Actives**	**Inactives**
**1**	6538P	13	48
**2**	25923	12	49
**3**	4S	12	49
**4**	5S	14	47
**5**	19S	14	47

a) dataset number used for the combination;
b) column indicating the number of samples as active or inactives.

Table SM11. List of hyperparameters setting used for the models' random search optimization.

Algorithm	Parameters	Settings	Total Combinations
RF	class_weight	list_weight	133970183
	n_estimators	from 1 to 200, step 1	
	max_depth	from 1 to 200, step 1	
	min_samples_leaf	from 1 to 200, step 1	
GB	n_estimators	from 1 to 200, step 1	7880599
	max_depth	from 1 to 200, step 1	
	min_samples_leaf	from 1 to 200, step 1	
LR	class_weight	list_weight	50745
	C	from 1 to 200, step 1	
	penalty	l1, l2, elasticnet	
	solver	newton-cg, lbfgs, liblinear, sag, saga	
	max_iter	10000	
SV	class_weight	list_weight	13532
	C	from 1 to 200, step 1	
	kernel	linear, poly, rbf, sigmoid	
	probability	True	
DT	class_weight	list_weight	2176000
	criterion	gini, entropy	
	splitter	best, random	
	max_depth	from 1 to 21, step 1	
	min_samples_split	from 1 to 21, step 1	
	min_samples_leaf	from 1 to 21, step 1	
	max_features	auto, sqrt, log2, None	
KNN	n_neighbors	from 1 to 31, step 1	57600
	weights	uniform, distance	
	algorithm	auto, ball_tree, kd_tree, brute	
	leaf_size	from 1 to 31, step 1	
	metric	minkowski, euclidean, manhattan, chebyshev	
	metric_params	None	
	p	1, 2	

Table SM12. Random forest models MCC and ROC AUC values.

#[a]	Strain	T[b]	A[a] Biofilm 0.05 % v/v[c]			B Biofilm 1.00 % v/v[d]			C Biofilm 1.00 % v/v Corr[e]		
			L[f]	MCC	AUC	L	MCC	AUC	L	MCC	AUC
**1**	6538P	40%	1	0.67	0.87	1	0.67	0.87	2	0.33	0.58
**2**	25923		1	0.83	0.92	1	0.83	0.92	4	0.19	0.85
**3**	4S		-[g]	-	-	-	-	-	2	0.27	0.49
**4**	5S		-	-	-	-	-	-	1	0.00[h]	0.00
**5**	19S		-	-	-	-	-	-	4	0.15	0.51
**6**	6538P	80%	3	0.68	0.82	3	0.68	0.82	1	0.50	0.72
**7**	25923		4	0.33	0.61	4	0.33	0.61	4	0.38	0.62
**8**	4S		1	0.14	0.69	1	0.14	0.69	1	0.00	0.00
**9**	5S		-	-	-	-	-	-	2	0.19	0.36
**10**	19S		-	-	-	-	-	-	1	0.18	0.27
**11**	6538P	100%	4	0.53	0.78	4	0.53	0.78	1	0.55	0.62
**12**	25923		1	0.26	0.50	1	0.26	0.50	1	0.20	0.59
**13**	4S		3	0.16	0.51	3	0.16	0.51	-	-	-
**14**	5S		1	0.46	0.66	1	0.46	0.66	2	0.12	0.47
**15**	19S		2	0.41	0.72	2	0.41	0.72	1	0.33	0.56
**16**	6538P	120%	1	0.37	0.69	1	0.37	0.69	1	0.55	0.65
**17**	25923		-	-	-	-	-	-	1	0.00	0.00
**18**	4S		1	0.26	0.39	1	0.26	0.39	-	-	-
**19**	5S		3	0.10	0.39	3	0.10	0.39	1	0.00	0.00
**20**	19S		3	0.25	0.65	3	0.25	0.65	-	-	-
**21**	6538P	median	2	0.32	0.66	2	0.32	0.66	1	0.17	0.55
**22**	25923		4	0.44	0.61	4	0.44	0.61	4	0.32	0.70
**23**	4S		1	0.19	0.62	1	0.19	0.62	2	0.14	0.49
**24**	5S		3	0.29	0.45	3	0.29	0.45	1	0.13	0.49
**25**	19S		2	0.28	0.68	2	0.28	0.68	1	0.00	0.00

a) dataset numbers and letters used for the dataset combination: 1A, 2A….1B, 2B….1C,2C…25C;
b) Threshold used to binarize data;
c) column indicating the MCC and ROC AUC values for datasets compiled with biofilm formation at 0.05% v/v concentration;
d) column indicating the MCC and ROC AUC values for datasets compiled with biofilm formation at 1.00% v/v concentration;
e) column indicating the MCC and ROC AUC values for datasets compiled with biofilm formation at 1.00% v/v concentration implemented with data at 0.05% v/v for missing values.
f) optimal nlevel value
g) a dash indicates too unbalanced dataset and no model was elaborated
h) 0.00 indicates the model was characterized with MCC negative values

Table SM13. Gradient bosting models MCC and ROC AUC values.

#[a]	Strain	T[b]	A[a] Biofilm 0.05 % v/v[c]			B Biofilm 1.00 % v/v[d]			C Biofilm 1.00 % v/v Corr[e]		
			L[f]	MCC	AUC	L	MCC	AUC	L	MCC	AUC
**1**	6538P	40%	4	0.88	0.93	4	0.88	0.93	1	0.24	0.66
**2**	25923		1	1.00	1.00	1	1.00	1.00	4	0.22	0.76
**3**	4S		-[g]	-	-	-	-	-	2	0.07	0.63
**4**	5S		-	-	-	-	-	-	3	0.25	0.67
**5**	19S		-	-	-	-	-	-	1	0.15	0.69
**6**	6538P	80%	2	0.75	0.89	2	0.75	0.89	1	0.52	0.81
**7**	25923		2	0.35	0.69	2	0.35	0.69	1	0.29	0.60
**8**	4S		4	0.25	0.64	4	0.25	0.64	1	0.00[h]	0.00
**9**	5S		-	-	-	-	-	-	4	0.36	0.72
**10**	19S		-	-	-	-	-	-	2	0.32	0.58
**11**	6538P	100%	1	0.53	0.84	1	0.53	0.84	1	0.57	0.68
**12**	25923		2	0.48	0.77	2	0.48	0.77	1	0.41	0.61
**13**	4S		2	0.32	0.59	2	0.32	0.59	-	-	-
**14**	5S		4	0.63	0.71	4	0.63	0.71	4	0.11	0.66
**15**	19S		1	0.38	0.67	1	0.38	0.67	3	0.58	0.82
**16**	6538P	120%	3	0.45	0.72	3	0.45	0.72	1	0.34	0.63
**17**	25923		-	-	-	-	-	-	4	0.21	0.39
**18**	4S		3	0.15	0.32	3	0.15	0.32	-	-	-
**19**	5S		1	0.00	0.00	1	0.00	0.00	1	0.30	0.44
**20**	19S		4	0.45	0.67	4	0.45	0.67	-	-	-
**21**	6538P	median	2	0.32	0.69	2	0.32	0.69	4	0.13	0.51
**22**	25923		2	0.18	0.64	2	0.18	0.64	1	0.31	0.66
**23**	4S		1	0.55	0.73	1	0.55	0.73	2	0.18	0.54
**24**	5S		1	0.00	0.00	1	0.00	0.00	1	0.28	0.61
**25**	19S		2	0.38	0.72	2	0.38	0.72	1	0.00	0.00

a) dataset numbers and letters used for the dataset combination: 1A, 2A….1B, 2B….1C,2C…25C;
b) Threshold used to binarize data;
c) column indicating the MCC and ROC AUC values for datasets compiled with biofilm formation at 0.05% v/v concentration;
d) column indicating the MCC and ROC AUC values for datasets compiled with biofilm formation at 1.00% v/v concentration;
e) column indicating the MCC and ROC AUC values for datasets compiled with biofilm formation at 1.00% v/v concentration implemented with data at 0.05% v/v for missing values.
f) optimal nlevel value
g) a dash indicates too unbalanced dataset and no model was elaborated
h) 0.00 indicates the model was characterized with MCC negative values

Table SM14. Logistic regression models MCC and ROC AUC values.

#[a]	Strain	T[b]	A[a]			B			C		
			Biofilm 0.05 % v/v[c]			Biofilm 1.00 % v/v[d]			Biofilm 1.00 % v/v Corr[e]		
			L[f]	MCC	AUC	L	MCC	AUC	L	MCC	AUC
**1**	6538P	40%	1	0.83	0.97	1	0.83	0.97	1	0.28	0.65
**2**	25923		3	1.00	1.00	3	1.00	1.00	4	0.14	0.58
**3**	4S		-[g]	-	-	-	-	-	2	0.09	0.52
**4**	5S		-	-	-	-	-	-	1	0.13	0.58
**5**	19S		-	-	-	-	-	-	1	0.00[h]	0.00
**6**	6538P	80%	2	0.73	0.91	2	0.73	0.91	1	0.73	0.84
**7**	25923		4	0.18	0.42	4	0.18	0.42	4	0.33	0.65
**8**	4S		1	0.36	0.71	1	0.36	0.71	4	0.06	0.52
**9**	5S		-	-	-	-	-	-	4	0.26	0.45
**10**	19S		-	-	-	-	-	-	1	0.26	0.45
**11**	6538P	100%	2	0.52	0.77	2	0.52	0.77	1	0.43	0.84
**12**	25923		1	0.27	0.65	1	0.27	0.65	1	0.38	0.68
**13**	4S		4	0.06	0.50	4	0.06	0.50	-	-	-
**14**	5S		4	0.57	0.70	4	0.57	0.70	2	0.25	0.49
**15**	19S		3	0.46	0.69	3	0.46	0.69	1	0.33	0.51
**16**	6538P	120%	2	0.41	0.61	2	0.41	0.61	1	0.43	0.86
**17**	25923		-	-	-	-	-	-	1	0.35	0.54
**18**	4S		1	0.08	0.44	1	0.08	0.44	-	-	-
**19**	5S		4	0.18	0.44	4	0.18	0.44	1	0.28	0.55
**20**	19S		2	0.50	0.78	2	0.50	0.78	-	-	-
**21**	6538P	median	2	0.28	0.66	2	0.28	0.66	4	0.35	0.57
**22**	25923		3	0.27	0.48	3	0.27	0.48	1	0.36	0.74
**23**	4S		4	0.20	0.59	4	0.20	0.59	1	0.11	0.48
**24**	5S		3	0.35	0.47	3	0.35	0.47	2	0.20	0.54
**25**	19S		2	0.35	0.65	2	0.35	0.65	1	0.06	0.47

a) dataset numbers and letters used for the dataset combination: 1A, 2A….1B, 2B….1C,2C…25C;
b) Threshold used to binarize data;
c) column indicating the MCC and ROC AUC values for datasets compiled with biofilm formation at 0.05% v/v concentration;
d) column indicating the MCC and ROC AUC values for datasets compiled with biofilm formation at 1.00% v/v concentration;
e) column indicating the MCC and ROC AUC values for datasets compiled with biofilm formation at 1.00% v/v concentration implemented with data at 0.05% v/v for missing values.
f) optimal nlevel value
g) a dash indicates too unbalanced dataset and no model was elaborated
h) 0.00 indicates the model was characterized with MCC negative values

Table SM15. Support vector machine models MCC and ROC AUC values.

#[a]	Strain	T[b]	A[a] Biofilm 0.05 % v/v[c]			B Biofilm 1.00 % v/v[d]			C Biofilm 1.00 % v/v Corr[e]		
			L[f]	MCC	AUC	L	MCC	AUC	L	MCC	AUC
**1**	6538P	40%	2	0.94	0.98	2	0.94	0.98	2	0.33	0.25
**2**	25923		1	1.00	1.00	1	1.00	1.00	3	0.05	0.17
**3**	4S		-[g]	-	-	-	-	-	3	0.33	0.51
**4**	5S		-	-	-	-	-	-	1	0.24	0.00
**5**	19S		-	-	-	-	-	-	4	0.28	0.43
**6**	6538P	80%	1	0.82	0.90	1	0.82	0.90	1	0.63	0.80
**7**	25923		2	0.34	0.60	2	0.34	0.60	4	0.25	0.55
**8**	4S		4	0.39	0.67	4	0.39	0.67	4	0.18	0.13
**9**	5S		-	-	-	-	-	-	3	0.33	0.35
**10**	19S		-	-	-	-	-	-	4	0.23	0.06
**11**	6538P	100%	3	0.64	0.76	3	0.64	0.76	1	0.57	0.63
**12**	25923		2	0.48	0.62	2	0.48	0.62	4	0.27	0.33
**13**	4S		2	0.09	0.10	2	0.09	0.10	-	-	-
**14**	5S		3	0.63	0.58	3	0.63	0.58	1	0.31	0.28
**15**	19S		4	0.27	0.24	4	0.27	0.24	1	0.47	0.03
**16**	6538P	120%	3	0.52	0.76	3	0.52	0.76	1	0.57	0.54
**17**	25923		-	-	-	-	-	-	1	0.09	0.22
**18**	4S		3	0.24	0.22	3	0.24	0.22	-	-	-
**19**	5S		2	0.21	0.39	2	0.21	0.39	3	0.33	0.48
**20**	19S		4	0.46	0.49	4	0.46	0.49	-	-	-
**21**	6538P	median	3	0.48	0.66	3	0.48	0.66	2	0.30	0.35
**22**	25923		2	0.35	0.38	2	0.35	0.38	4	0.34	0.51
**23**	4S		3	0.25	0.55	3	0.25	0.55	2	0.24	0.21
**24**	5S		3	0.26	0.44	3	0.26	0.44	4	0.25	0.45
**25**	19S		4	0.32	0.39	4	0.32	0.39	1	0.15	0.42

a) dataset numbers and letters used for the dataset combination: 1A, 2A….1B, 2B….1C,2C…25C;
b) Threshold used to binarize data;
c) column indicating the MCC and ROC AUC values for datasets compiled with biofilm formation at 0.05% v/v concentration;
d) column indicating the MCC and ROC AUC values for datasets compiled with biofilm formation at 1.00% v/v concentration;
e) column indicating the MCC and ROC AUC values for datasets compiled with biofilm formation at 1.00% v/v concentration implemented with data at 0.05% v/v for missing values.
f) optimal nlevel value
g) a dash indicates too unbalanced dataset and no model was elaborated

Table SM16. Decision trees models MCC and ROC AUC values.

#[a]	Strain	T[b]	A[a] Biofilm 0.05 % v/v[c]			B Biofilm 1.00 % v/v[d]			C Biofilm 1.00 % v/v Corr[e]		
			L[f]	MCC	AUC	L	MCC	AUC	L	MCC	AUC
**1**	6538P	40%	2	0.83	0.92	2	0.83	0.92	4	0.42	0.41
**2**	25923		2	0.93	0.98	2	0.93	0.98	4	0.54	0.69
**3**	4S		-[g]	-	-	-	-	-	2	0.38	0.48
**4**	5S		-	-	-	-	-	-	3	0.42	0.65
**5**	19S		-	-	-	-	-	-	4	0.31	0.55
**6**	6538P	80%	2	0.80	0.89	2	0.80	0.89	2	0.62	0.77
**7**	25923		4	0.61	0.78	4	0.61	0.78	4	0.52	0.77
**8**	4S		1	0.44	0.62	1	0.44	0.62	2	0.05	0.36
**9**	5S		-	-	-	-	-	-	4	0.47	0.50
**10**	19S		-	-	-	-	-	-	2	0.42	0.64
**11**	6538P	100%	2	0.60	0.79	2	0.60	0.79	4	0.57	0.84
**12**	25923		1	0.39	0.62	1	0.39	0.62	1	0.55	0.37
**13**	4S		2	0.24	0.50	2	0.24	0.50	-	-	-
**14**	5S		4	0.68	0.74	4	0.68	0.74	4	0.45	0.56
**15**	19S		1	0.44	0.67	1	0.44	0.67	3	0.61	0.80
**16**	6538P	120%	4	0.51	0.46	4	0.51	0.46	4	0.74	0.83
**17**	25923		-	-	-	-	-	-	1	0.38	0.41
**18**	4S		2	0.11	0.48	2	0.11	0.48	-	-	-
**19**	5S		3	0.36	0.48	3	0.36	0.48	2	0.35	0.35
**20**	19S		3	0.41	0.72	3	0.41	0.72	-	-	-
**21**	6538P	median	1	0.36	0.56	1	0.36	0.56	4	0.37	0.45
**22**	25923		1	0.29	0.53	1	0.29	0.53	1	0.51	0.61
**23**	4S		1	0.55	0.76	1	0.55	0.76	2	0.39	0.54
**24**	5S		3	0.38	0.41	3	0.38	0.41	2	0.36	0.56
**25**	19S		2	0.43	0.73	2	0.43	0.73	4	0.08	0.38

a) dataset numbers and letters used for the dataset combination: 1A, 2A….1B, 2B….1C,2C…25C;
b) Threshold used to binarize data;
c) column indicating the MCC and ROC AUC values for datasets compiled with biofilm formation at 0.05% v/v concentration;
d) column indicating the MCC and ROC AUC values for datasets compiled with biofilm formation at 1.00% v/v concentration;
e) column indicating the MCC and ROC AUC values for datasets compiled with biofilm formation at 1.00% v/v concentration implemented with data at 0.05% v/v for missing values.
f) optimal nlevel value
g) a dash indicates too unbalanced dataset and no model was elaborated

Table SM17. KNN models MCC and ROC AUC values.

#[a]	Strain	T[b]	A[a] Biofilm 0.05 % v/v[c]			B Biofilm 1.00 % v/v[d]			C Biofilm 1.00 % v/v Corr[e]		
			L[f]	MCC	AUC	L	MCC	AUC	L	MCC	AUC
**1**	6538P	40%	4	0.88	0.93	4	0.88	0.93	3	0.41	0.70
**2**	25923		1	1.00	1.00	1	1.00	1.00	4	0.26	0.62
**3**	4S		-	-	-	-	-	-	1	0.38	0.39
**4**	5S		-	-	-	-	-	-	4	0.18	0.57
**5**	19S		-	-	-	-	-	-	4	0.29	0.70
**6**	6538P	80%	1	0.77	0.88	1	0.77	0.88	1	0.29	0.71
**7**	25923		3	0.27	0.58	3	0.27	0.58	3	0.13	0.58
**8**	4S		1	0.39	0.76	1	0.39	0.76	1	0.17	0.52
**9**	5S		-	-	-	-	-	-	3	0.11	0.39
**10**	19S		-	-	-	-	-	-	1	0.16	0.39
**11**	6538P	100%	1	0.61	0.79	1	0.61	0.79	1	0.39	0.54
**12**	25923		2	0.42	0.68	2	0.42	0.68	1	0.10	0.49
**13**	4S		3	0.23	0.40	3	0.23	0.40	-	-	-
**14**	5S		4	0.52	0.77	4	0.52	0.77	3	0.23	0.62
**15**	19S		3	0.39	0.62	3	0.39	0.62	1	0.25	0.55
**16**	6538P	120%	1	0.59	0.76	1	0.59	0.76	1	0.39	0.58
**17**	25923		-	-	-	-	-	-	1	0.21	0.53
**18**	4S		2	0.27	0.35	2	0.27	0.35	-	-	-
**19**	5S		2	0.31	0.56	2	0.31	0.56	1	0.20	0.57
**20**	19S		2	0.37	0.67	2	0.37	0.67	-	-	-
**21**	6538P	median	1	0.44	0.68	1	0.44	0.68	3	0.38	0.64
**22**	25923		1	0.21	0.57	1	0.21	0.57	3	0.31	0.56
**23**	4S		3	0.31	0.56	3	0.31	0.56	4	0.20	0.40
**24**	5S		2	0.35	0.52	2	0.35	0.52	2	0.28	0.64
**25**	19S		3	0.35	0.56	3	0.35	0.56	1	0.00	0.00

a) dataset numbers and letters used for the dataset combination: 1A, 2A….1B, 2B….1C,2C…25C;
b) Threshold used to binarize data;
c) column indicating the MCC and ROC AUC values for datasets compiled with biofilm formation at 0.05% v/v concentration;
d) column indicating the MCC and ROC AUC values for datasets compiled with biofilm formation at 1.00% v/v concentration;
e) column indicating the MCC and ROC AUC values for datasets compiled with biofilm formation at 1.00% v/v concentration implemented with data at 0.05% v/v for missing values.
f) optimal nlevel value
g) a dash indicates too unbalanced dataset and no model was elaborated

Table SM18. RF, GB, LR, SV, DT and KNN ML learning algorithms MCC and ROC AUC values for the antimicrobial dataset.

#[a]	Strain[b]	RF[c]			GB[c]			LR[c]			SV[c]			DT[c]			KNN[c]		
		L[d]	MCC[e]	AUC[f]	L	MCC	AUC	L	MCC	AUC	L	MCC	AUC	L	MCC	AUC	L	MCC	AUC
**1D**	6538P	1	0.44	0.80	4	0.57	0.86	1	0.44	0.80	4	0.69	0.85	1	0.63	0.88	3	0.65	0.84
**2D**	25923	1	0.46	0.82	2	0.54	0.82	1	0.46	0.82	4	0.72	0.86	3	0.60	0.82	2	0.63	0.86
**3D**	4S	4	0.47	0.80	2	0.54	0.84	4	0.47	0.80	4	0.72	0.87	3	0.66	0.80	2	0.67	0.87
**4D**	5S	4	0.71	0.89	4	0.76	0.87	4	0.71	0.89	2	0.95	0.99	2	0.71	0.83	2	0.87	0.93
**5D**	19S	4	0.65	0.85	3	0.71	0.77	4	0.65	0.85	4	0.81	0.93	2	0.71	0.87	2	0.76	0.91

a) Dataset combination defined similarly as from tables 12-17; b) *S aureus* strain code; c) Machine learning agorithm as defined in Material and Methods; ; d) Matthew correlation coefficient; e) ROC AUC values.

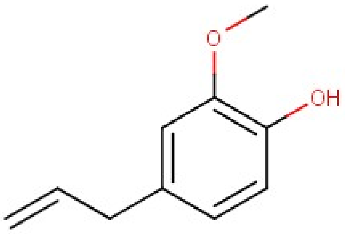

eugenol

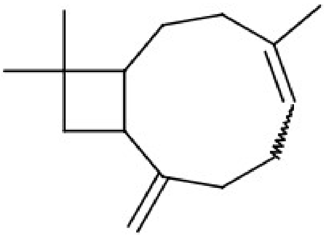

β-caryophyllene

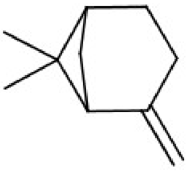

□-pinene

Figure SM1. Chemical structure of compounds mostly predicted as reducer of biofilm production (main text Table 3).

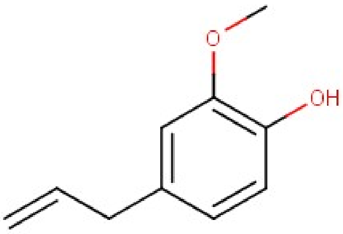

eugenol

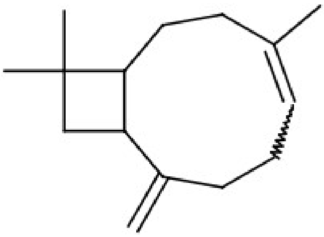

β-caryophyllene

Figure SM2. Chemical structure of compounds predicted to have both ability to reduce biofilm production and as antibacterial (main text Table 3).

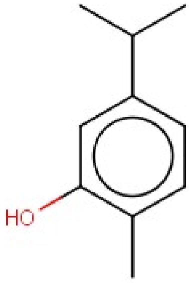

carvacrol

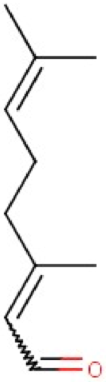

□-citral

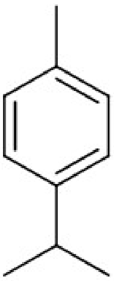

p-cymene

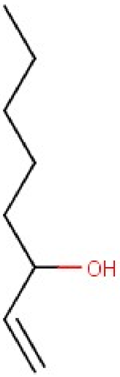

1-octen-3-ol

Figure SM3. Chemical structure of compounds predicted to increase antibacterial potency (main text Table 3).

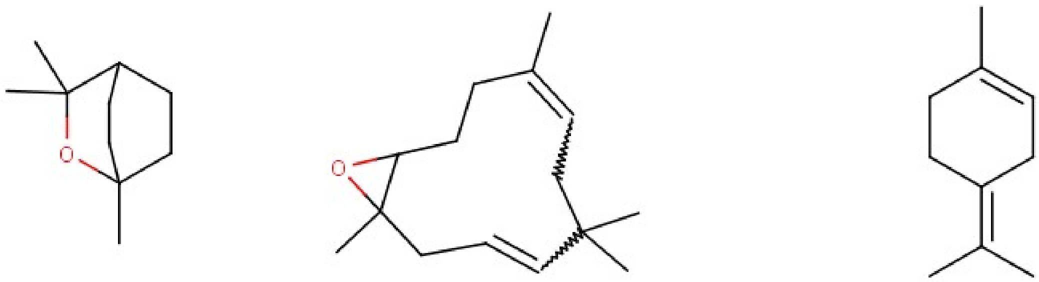

eucalyptol humulene epoxide 2 terpinolene

Figure SM4. Example of chemical structure of compounds predicted to have the ability to likely modulate the inducing of biofilm production or counteracting biofilm inhibition (main text Table 3).

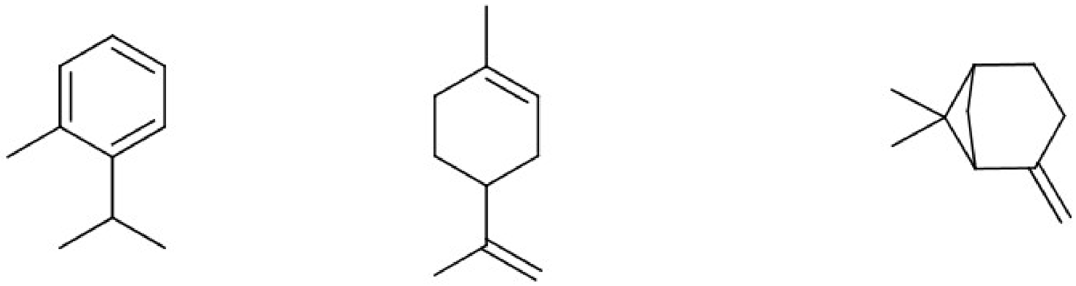

o-cymene limonene □-pinene

Figure SM5. Chemical structure of compounds predicted not to have fixed behavior on different *S. aureus* bacterial strains (main text Table 3).

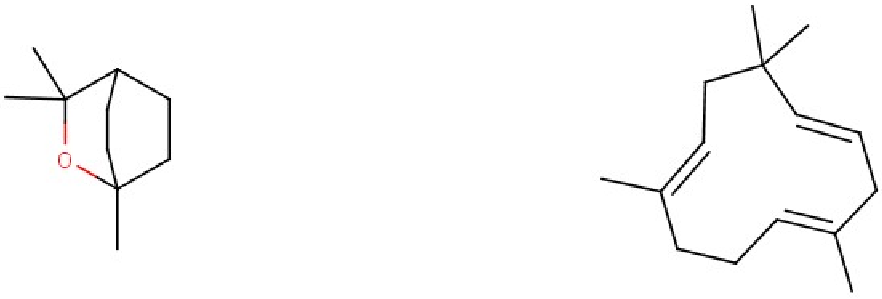

eucalyptol humulene

Figure SM6. Chemical structure of compounds predicted to have ability to induce biofilm production and antisynergistic antibacterial activity (main text Table 3).

Table SM18. Compositions of the 61 essential oils used in the study

	EO1
.τ.-muurolol	2.1
artemisia alcohol	1.3
borneol	1.2
borneol, butyrate	4.3
β-caryophyllene	1.0
caryophyllene oxide	1.3
cis-3-pinanone	0.3
cis-β-farnesene	5.2
eucalyptol	2.0
germacrene d	4.5
isobornyl 3-methylbutanoate	3.1
limonene	6.7
linalool	0.3
myrtenol	0.4
o-cymene	0.2
pinocarveol	7.4
sativene	0.9
spathulenol	4.0
terpinen-4-ol	0.4
trans-2,7-dimethyl-4,6-octadien-2-ol	33.2
yomogi alcohol	1.3
α-pinene	14.4
β-myrcene	1.4
δ-cadinene	0.5
δ-elemene	1.7
τ-cadinol	0.5

	EO2
β-caryophyllene	2.0
caryophyllene oxide	2.3
cis-geraniol	1.7
geranyl acetate	3.5
linalool	19.9
linalyl anthranilate	60.0
nerol acetate	2.2
trans-linalool oxide	2.1
α-bergamotene	0.8
α-copaene	0.6
α-terpineol	3.8
β-bisabolene	0.7
β-myrcene	0.3

	EO3
alcanfor	21.3
borneol	4.6
bornyl acetate	2.6
camphene	3.5
β-caryophyllene	6.8
caryophyllene oxide	0.3
chrysanthone	25.1
eucalyptol	11.7
humulene	7.5
humulene epoxide 2	0.3
limonene	1.3
o-cymene	1.3
terpinen-4-ol	0.6
thujone	7.8
viridiflorol	0.2
α-pinene	3.0
β-pinene	1.4
γ-terpinene	0.5

	EO4
borneol	1.5
carvacrol	7.2
β-caryophyllene	2.3
caryophyllene oxide	0.6
eucalyptol	0.3
limonene	0.3
linalool	5.2
methyl thymyl ether	0.2
p-cymene	10.5
terpinen-4-ol	1.8
thymol	66.3
α-citral	0.1
α-pinene	0.4
α-terpinene	0.4
α-terpineol	0.2
β-myrcene	0.5
γ-terpinene	2.3

	EO5
eucalyptol	14.9
globulol	0.4
limonene	2.0
linalool, oxide	0.2
longifolene	0.2
o-cymene	3.5
terpinen-4-ol	37.5
terpinolene	1.7
viridiflorene	1.1
α-gurjunene	0.2
α-pinene	11.1
α-terpinene	4.6
α-terpineol	8.1
β-myrcene	0.2
β-pinene	2.5
γ-terpinene	11.8

	EO6
6-methyl-5-hepten-2-one	0.3
β-caryophyllene	31.4
caryophyllene oxide	5.5
cis-geraniol	2.5
citronellal	26.3
eucalyptol	0.7
humulene epoxide 2	0.9
limonene	1.6
linalool	0.8
α-citral	28.2
α-copaene	0.7
γ-cadinene	0.6
δ-cadinene	0.5

	EO7
3-carene	10.8
bornyl acetate	13.4
calamenene	0.7
carvone	0.3
β-caryophyllene	21.4
cis-verbenol	0.5
crypton	3.7
cumaldehyde	0.5
humulene	1.2
isopinocarveol	0.5
limonene	10.9
linalool	0.2
o-cymene	2.1
p-cymen-8-ol	0.8
trans-2-caren-4-ol	0.6
α-copaene	0.9
α-muurolene	1.1
α-pinene	12.5
α-terpineol	0.4
β-cubebene	1.2
β-phellandrene	7.0
β-pinene	7.6
δ-cadinene	1.6

	EO8
1-indanone	3.8
2,6-dimethyl-2,6-octadiene	0.6
2-pentadecanone, 6,10,14-trimethyl	0.4
acetic acid	5.0
cis-geraniol	12.9
citronellol	26.3
citronellyl butyrate	1.5
citronellyl formate	8.0
geraniol formate	3.8
geranyl isobutyrate	1.2
geranyl propionate	0.8
heptanoic acid	1.2
hexanoic acid	0.6
isomenthone	4.6
limonene	8.4
linalool	9.6
linalyl anthranilate	6.8
o-cymene	1.3
rose oxide	0.9
α-pinene	0.5
β-myrcene	0.4
β-pinene	1.2

	EO9
borneol	0.4
carvacrol	76.5
β-caryophyllene	6.7
caryophyllene oxide	2.3
eucalyptol	0.4
limonene	0.5
linalool	2.4
p-cymene	6.8
terpinen-4-ol	0.5
α-pinene	0.4
α-terpinene	0.3
β-myrcene	0.8
γ-terpinene	1.9

	EO10
acetic acid, cinnamyl ester	1.0
calamenene	0.3
β-caryophyllene	15.5
cis-geraniol	1.5
cis-β-farnesene	13.9
eucalyptol	0.2
eugenol	0.6
farnesol	2.6
farnesyl acetate	3.5
geranyl acetate	12.2
germacrene d	18.2
humulene	4.3
linalool	10.4
methyl benzoate	2.1
p-methylanisole	3.3
α-cadinol	1.1
α-copaene	1.2
β-elemene	0.5
β-ylangene	0.5
γ-cadinene	1.0
γ-muurolene	1.8
δ-cadinene	3.8
τ-cadinol	0.6

	EO11
3,7-octadiene-2,6-diol, 2,6-dimethyl-	0.2
benzyl benzoate	6.4
camphene	0.4
camphor	5.7
cis-geraniol	1.7
eucalyptol	0.4
geranyl acetate	5.2
limonene	1.7
linalool	66.7
o-cymene	2.5
salicyclic acid, benzyl ester	2.2
terpinen-4-ol	0.2
trans-linalool oxide	0.6
α-pinene	3.4
α-terpineol	0.8
β-myrcene	0.5
β-pinene	0.3
γ-terpinene	0.9

	EO12
1-octen-3-ol	0.3
3-octanol	0.3
3-octanone	0.9
borneol	1.4
butanoic acid, hexyl ester	0.5
β-caryophyllene	2.7
cis-geraniol	0.4
cis-β-farnesene	1.9
cis-β-ocimene	1.0
eucalyptol	2.4
lavandulyl acetate	4.3
limonene	0.5
linalool	34.7
linalyl acetate	41.4
terpinen-4-ol	3.8
α-pinene	0.2
α-terpineol	1.3
β-myrcene	0.7
β-ocimene	1.3

	EO13
1,4-dihydroxy-p-menth-2-ene	0.3
3-carene	0.2
β-caryophyllene	2.2
cis-geraniol	0.7
eucalyptol	40.8
geranyl acetate	9.7
humulene	0.5
isobutyl 2-methylbutyrate	0.5
isobutyl isobutyrate	0.3
limonene	7.1
linalool	3.7
linalyl anthranilate	1.2
methyleugenol	2.0
o-cymene	5.3
p-cymen-8-ol	0.7
pinocarveol	0.2
terpinen-4-ol	0.6
α-pinene	17.4
α-terpineol	3.9
α-terpineol acetate	1.9
β-myrcene	0.2
β-pinene	0.2
γ-elemene	0.2

	EO14
1,2-dithiolane	0.5
1,2-dithiolane, 1,1-dioxide	2.8
3-vinyl-1,2-dithiacyclohex-4-ene	2.6
diallyl disulphide	57.6
diallyl sulfide	3.8
dimethyl trisulfide	0.5
disulfide, methyl 2-propenyl	2.7
eucalyptol	0.3
limonene	0.5
trans-3,5-diethyl-1,2,4-trithiolane	0.3
trisulfide, di-2-propenyl	2.1
trisulfide, methyl 2-propenyl	26.1

	EO15
cis-geraniol	1.1
cis-β-terpineol	0.7
eucalyptol	34.7
limonene	2.0
linalool	5.0
linalyl anthranilate	8.2
nerolidol	1.3
o-cymene	0.4
sabinene	0.3
α-pinene	1.3
α-terpineol	1.9
α-terpineol acetate	41.8
β-myrcene	1.1
β-pinene	0.3

	EO16
limonene	66.7
o-cymene	7.6
sabinene	0.2
terpinolene	0.9
α-pinene	2.4
α-terpineol	0.3
β-myrcene	1.6
β-pinene	1.3
γ-terpinene	19.0

	EO17
4-methoxycinnamaldehyde	1.1
borneol	1.1
β-caryophyllene	1.3
caryophyllene oxide	0.6
cis-3-pinanone	34.1
dhs activator	0.6
elemol	1.1
estragole	0.2
eucalyptol	0.6
limonene	1.4
linalool	18.0
myrtenal	0.5
myrtenol	1.0
o-cymene	0.5
pinocarveol	0.3
sabinene	0.6
spathulenol	1.1
terpinen-4-ol	0.9
trans-pinocamphone	18.9
α-pinene	8.6
α-terpineol	0.8
β-myrcene	0.3
β-pinene	6.4

	EO18
p-mentha-2,8-dien-1-ol	1.9
carveol	1.9
carvone	4.7
caryophyllene oxide	0.4
cis-carveol	3.3
cis-limonene oxide	3.5
limonene	78.2
limonene-1,2-diol	0.7
o-cymene	0.3
trans-limonene oxide	2.4
trans-p-mentha-2,8-dienol	2.1
α-pinene	0.4
β-myrcene	0.3

	EO19
.τ.-muurolol	0.2
β-caryophyllene	0.2
citronellal	38.8
citronellol	19.0
citronellyl acetate	2.9
elemol	2.5
eugenol	0.9
geraniol	24.8
germacrene D	0.7
germacrene-D-4-ol	0.3
isopulegol	0.7
linalool	0.4
α-cadinol	0.4
α-citral	2.0
β-citral	1.2
β-elemene	0.9
β-myrcene	3.0
γ-muurolene	0.3
δ-cadinene	0.9

	EO20
borneol	2.7
bornyl acetate	55.0
camphene	18.1
camphor	0.4
β-caryophyllene	0.9
caryophyllene oxide	1.3
crypton	0.6
humulene	0.5
humulene epoxide 2	0.6
limonene	3.9
o-cymene	0.4
p-cymen-7-ol	0.2
p-cymen-8-ol	0.7
santene	1.0
tricyclene	1.5
α-bisabolol	0.4
α-pinene	8.4
α-terpineol	0.4
β-bisabolene	0.2
β-phellandrene	1.1
β-pinene	1.6

	EO21
1,4-dihydroxy-p-menth-2-ene	0.5
camphene	0.4
estragole	0.3
eucalyptol	57.0
limonene	22.8
m-cymene	13.4
palmitic acid	1.6
sabinene	0.6
α-pinene	0.4
β-pinene	3.1

	EO22
2,2,7,7-tetramethyltricyclo[6.2.1.0~1,6~]undeca-3,5,9-triene	2.4
2-methoxy-4-propylphenol	3.0
2-methoxy-phenol	4.2
2-methyl-phenol	1.3
3-methyl-1,2-cyclopentanedione	1.2
4-ethyl-2-methoxyphenol	7.3
8,14-cedranoxide	0.3
calamenene	17.4
β-caryophyllene	1.0
cedrene	8.8
cedrol	0.5
creosol	9.3
cubenol	2.7
humulene	1.6
isoeugenol	1.9
isoledene	3.7
m-cresol	1.4
phenol	1.0
α-cadinol	0.5
α-calacorene	6.3
α-curcumene	1.1
α-muurolene	4.6
δ-cadinene	18.6

	EO23
bornyl acetate	0.6
camphene	0.3
camphor	3.8
chrysanthone	88.5
eucalyptol	0.3
fenchone	2.7
limonene	0.3
sabinene	0.3
terpinen-4-ol	0.7
thujone	1.8
α-pinene	0.4
β-myrcene	0.3

	EO24
6-methyl-5-hepten-2-one	0.6
borneol	1.1
camphene	5.7
cis-geraniol	0.4
elemol	0.5
eucalyptol	7.0
limonene	1.1
linalool	0.3
nerolidol	0.6
zingiberene	29.9
α-bergamotene	0.4
α-copaene	0.6
α-curcumene	31.6
α-farnesene	4.3
α-pinene	1.7
α-terpineol	0.7
β-bisabolene	10.6
β-elemene	1.2
β-eudesmol	0.3
β-myrcene	0.6
γ-muurolene	0.7

	EO25
2-caren-10-ol	28.1
carotol	1.3
carvacrol	0.3
β-caryophyllene	0.7
caryophyllene oxide	0.5
cis-β-farnesene	0.9
cumaldehyde	37.9
limonene	0.3
p-cymen-7-ol	0.5
p-cymen-8-ol	0.2
p-cymene	7.6
sabinene	0.2
α-phellandrene	1.0
α-pinene	0.3
β-myrcene	0.4
β-phellandrene	0.3
β-pinene	5.6
γ-terpinene	13.9

	EO26
β-caryophyllene	3.2
caryophyllene oxide	0.6
globulol	1.1
ledene oxide-(I)	0.4
patchouli alcohol	45.4
seychellene	6.8
α-guaiene	15.3
α-panasinsene	0.3
α-patchoulene	5.9
β-guaiene	19.0
β-patchoulene	2.1

	EO27
decanal	0.3
limonene	95.1
linalool	0.4
linalyl acetate	0.6
sabinene	0.2
α-pinene	0.5
β-pinene	2.7
γ-terpinene	0.1

	EO28
carvone	0.3
eucalyptol	83.9
limonene	6.1
p-cymene	8.1
α-pinene	1.1
β-myrcene	0.3
β-pinene	0.2

	EO29
3-carene	16.6
bornyl acetate	1.4
camphene	0.9
β-caryophyllene	16.7
caryophyllene oxide	3.5
cis-verbenol	0.2
humulene	1.2
humulene epoxide 2	0.3
limonene	11.2
longifolene	2.0
myrtenol	0.2
o-cymene	0.5
p-cymen-8-ol	1.6
pinocarveol	0.4
terpinolene	2.0
α-cubebene	0.5
α-pinene	22.8
α-terpineol	0.9
β-pinene	17.1

	EO30
carvone	0.4
cis-carveol	0.3
limonene	26.1
limonene oxide, cis-	0.3
linalool	13.8
linalyl anthranilate	45.3
nerol acetate	0.7
o-cymene	6.5
sabinene	0.5
α-pinene	0.7
β-bisabolene	0.2
β-myrcene	0.7
β-pinene	4.6

	EO31
(E)-β-farnesene	0.6
4-epi-cubebol	1.1
β-caryophyllene	13.7
caryophyllene oxide	2.9
humulene	3.6
limonene	7.5
o-cymene	3.0
p-cymen-8-ol	0.6
sabinene	5.9
spathulenol	0.8
terpinen-4-ol	6.3
terpinolene	0.9
trans-calamenene	0.9
α-cadinol	0.4
α-copaene	1.0
α-cubebene	0.9
α-muurolene	0.8
α-pinene	27.1
α-terpineol	0.6
β-elemene	3.6
β-myrcene	9.3
β-pinene	3.6
γ-elemene	2.0
γ-muurolene	1.5
γ-terpinene	1.0
δ-cadinene	0.4

	EO32
1,8-dimethylnaphthalene	4.6
2,2,7,7-tetramethyltricyclo[6.2.1.0~1,6~]undeca-3,5,9-triene	1.7
2-methoxy-4-propylphenol	2.7
2-methoxy-phenol	6.6
2-methyl-phenol	1.2
3-methyl-1,2-cyclopentanedione	0.8
4-epi-cubebol	2.8
benzocycloheptatriene	0.9
cadalene	5.5
cadina-1(2),4-diene	2.7
calamenene	18.1
cedrene	8.2
creosol	7.2
dihydrocurcumene	3.4
isoeugenol	1.6
m-cresol	1.3
α-cadinol	0.6
α-calacorene	5.8
α-muurolene	4.4
β-cadinene	0.7
δ-cadinene	18.5
δ-cadinol	0.8

	EO33
anethole	78.1
camphor	0.2
estragole	2.2
fenchone	3.4
limonene	3.4
o-cymene	0.4
p-acetonylanisole	4.7
p-anisaldehyde	6.3
α-bergamotene	0.1
α-pinene	1.0
β-myrcene	0.2

	EO34
carveol	0.4
carvone	1.4
β-caryophyllene	0.2
caryophyllene oxide	0.4
cis-p-mentha-2,8-dien-1-ol	0.4
geranic acid	1.0
limonene	32.2
limonene oxide, cis-	0.7
limonene oxide, trans-	0.5
linalool	6.4
linalyl anthranilate	25.0
nerol acetate	1.6
o-cymene	17.4
sabinene	0.2
trans-p-mentha-2,8-dienol	0.4
α-bergamotene	0.3
α-citral	4.8
α-pinene	0.4
β-bisabolene	0.5
β-citral	2.1
β-myrcene	0.4
β-pinene	3.2

	EO35
carveol	0.3
caryophyllene oxide	0.4
cis-geraniol	2.2
cis-p-mentha-2,8-dien-1-ol	0.4
geranic acid	1.6
geranyl acetate	1.0
limonene	59.2
limonene glycol	2.9
limonene oxide, trans-	0.7
myrtenal	0.2
nerol acetate	0.9
o-cymene	7.9
sabinene	1.0
α-citral	7.8
α-pinene	1.1
β-citral	3.3
β-myrcene	0.7
β-pinene	7.2
γ-terpinene	1.1

	EO36
artemisia alcohol	1.7
borneol	0.8
borneol, butyrate	1.7
bornyl acetate	1.0
camphene	0.4
β-caryophyllene	6.0
caryophyllene oxide	0.7
cis-3-pinanone	0.3
eucalyptol	2.5
germacrene d	1.8
limonene	7.3
o-cymene	0.2
pinocarveol	4.8
pinocarvone	0.3
sabinene	0.4
sativene	0.7
spathulenol	1.3
terpinen-4-ol	0.3
terpinolene	0.2
trans-2,7-dimethyl-4,6-octadien-2-ol	49.3
yomogi alcohol	1.3
α-pinene	18.9
β-myrcene	1.7
δ-elemene	1.5

	EO37
1-octen-3-ol	0.9
borneol	1.0
carvacrol	71.5
carvacrol acetate	0.3
β-caryophyllene	5.0
caryophyllene oxide	1.0
eucalyptol	0.4
limonene	0.2
linalool	1.5
p-cymene	5.6
spathulenol	0.3
terpinen-4-ol	1.1
thymol	1.6
α-pinene	0.3
α-terpinene	0.5
α-terpineol	0.2
β-bisabolene	4.1
β-myrcene	0.3
γ-cadinene	0.4
γ-muurolene	0.6
γ-terpinene	2.3
δ-cadinene	0.8

	EO38
borneol	1.3
bornyl acetate	0.4
camphene	0.8
camphor	21.4
β-caryophyllene	6.2
eucalyptol	53.2
eugenol	0.7
limonene	1.7
o-cymene	0.4
terpinen-4-ol	0.4
α-pinene	6.7
α-terpineol	1.2
β-myrcene	0.3
β-pinene	5.3

	EO39
2-methoxycinnamaldehyde	0.2
acetic acid, cinnamyl ester	2.6
acetyleugenol	1.3
β-caryophyllene	4.0
caryophyllene oxide	0.2
eugenol	34.6
humulene	0.6
limonene	0.2
linalool	3.2
o-cymene	0.7
tetradecanal	0.5
trans-3-phenyl-2-propenal	49.1
α-copaene	0.5
α-phellandrene	0.3
α-pinene	0.2
α-terpineol	0.3
β-isosafrole	0.9
β-phellandrene	0.5

	EO40
carvone	0.5
cis-carveol	0.2
eucalyptol	85.9
limonene	4.5
o-cymene	8.3
α-pinene	0.6

	EO41
carvone	0.8
cis-carveol	0.5
cis-p-mentha-2,8-dien-1-ol	0.3
limonene	95.0
linalool	0.8
sabinene	0.2
trans-p-mentha-2,8-dienol	0.3
α-pinene	0.4
β-myrcene	1.7

	EO42
β-caryophyllene	1.8
eucalyptol	66.0
ledol	5.4
limonene	5.9
linalool	0.2
o-cymene	1.5
terpinen-4-ol	0.9
terpinolene	0.4
α-pinene	5.3
α-terpineol	8.3
α-terpineol acetate	1.4
β-myrcene	0.7
β-pinene	1.5
γ-terpinene	0.8

	EO43
artemisia alcohol	2.0
borneol	0.9
camphene	3.0
camphor	27.0
cumaldehyde	0.4
eucalyptol	2.0
germacrene D	0.3
myrtenol	0.3
o-cymene	1.2
pinocarveol	0.7
sabinene	0.9
santolina triene	2.2
terpinen-4-ol	2.5
thujone	44.6
yomogi alcohol	1.6
α-pinene	0.2
β-thujone	9.9
γ-terpinene	0.3

	EO44
β-caryophyllene	9.1
cis-β-terpineol	0.7
eucalyptol	52.7
limonene	3.6
linalool	2.2
p-cymene	18.4
p-menth-3-en-1-ol	0.3
terpinolene	0.5
α-pinene	1.5
α-terpineol	7.6
α-terpineol acetate	1.2
β-myrcene	0.6
β-pinene	0.8
γ-terpinene	0.9

	EO45
3-carene	5.9
calamenene	0.3
β-caryophyllene	33.6
caryophyllene oxide	9.7
humulene	2.4
humulene epoxide 2	0.5
limonene	11.1
linalool	0.6
p-cymene	1.1
sabinene	7.2
spathulenol	0.4
terpinen-4-ol	0.4
α-bergamotene	0.1
α-copaene	5.4
α-muurolene	0.4
α-pinene	6.9
β-bisabolene	2.0
β-cubebene	0.7
β-elemene	0.7
β-pinene	6.3
β-selinene	0.5
γ-muurolene	0.2
δ-cadinene	0.9
δ-elemene	2.7

	EO46
1-octen-3-ol	0.5
borneol	2.2
camphene	1.0
camphor	2.4
carvacrol	6.6
β-caryophyllene	2.4
caryophyllene oxide	0.7
cis-β-terpineol	0.2
eucalyptol	1.2
limonene	0.5
linalool	7.5
methyl thymyl ether	0.4
p-cymene	18.2
terpinen-4-ol	3.0
thymol	44.4
α-pinene	1.3
α-terpinene	0.6
α-terpineol	0.2
β-myrcene	1.1
β-pinene	0.3
γ-terpinene	5.2

	EO47
1,4-dihydroxy-p-menth-2-ene	0.8
β-caryophyllene	0.6
caryophyllene oxide	1.0
cis-sabinene hydrate	2.9
cis-β-terpineol	15.4
eucalyptol	1.0
limonene	0.8
linalol oxide	0.5
linalool	40.7
o-cymene	8.5
p-cymen-8-ol	0.6
sabinene	2.6
terpinen-4-ol	23.2
α-pinene	0.4
α-terpineol	0.4
β-myrcene	0.5

	EO48
acetyleugenol	9.9
β-caryophyllene	8.8
eugenol	80.1
humulene	1.0
methylsalicylate	0.2

	EO49
3-carene	29.3
camphene	0.6
cedrene	0.5
cedrol	4.7
cis-verbenol	0.3
eucalyptol	0.5
limonene	4.0
linalool	0.7
o-cymene	1.4
p-cymen-8-ol	1.2
sabinene	0.7
terpinen-4-ol	1.5
terpinolene	0.7
trans-2-caren-4-ol	0.4
verbenone	0.3
α-pinene	47.7
α-terpineol	0.4
α-terpineol acetate	3.8
β-pinene	1.3

	EO50
eucalyptol	2.9
eugenol	2.2
geranyl acetate	0.2
isoeugenol	0.3
limonene	7.8
linalool	0.5
methyleugenol	0.3
myristicin	21.9
o-cymene	1.6
sabinene	13.9
terpinen-4-ol	16.6
terpinolene	0.7
α-copaene	0.3
α-phellandrene	0.8
α-pinene	12.0
α-terpinene	0.6
α-terpineol	0.7
α-terpineol acetate	0.1
β-isosafrole	1.5
β-pinene	11.1
γ-terpinene	3.8

	EO51
β-caryophyllene	0.2
eucalyptol	0.9
isomenthone	10.0
isopulegol	0.9
levomenthol	0.4
limonene	0.5
menthol	57.4
menthol, acetate	5.0
menthone	23.2
piperitenone	0.7
pulegone	0.4
α-terpineol	0.3

	EO52
6-methyl-5-hepten-2-one	1.3
β-caryophyllene	0.7
cis-geraniol	0.6
cis-verbenol	0.3
citronellal	8.0
citronellyl formate	1.1
elemol	0.5
eucalyptol	0.6
geranic acid	0.7
geraniol	5.6
limonene	9.5
linalool	1.3
sabinene	0.5
terpinen-4-ol	0.2
α-citral	33.7
α-copaene	0.1
α-pinene	1.6
β-citral	30.9
β-myrcene	0.4
β-pinene	2.1
δ-cadinene	0.2

	EO53
β-caryophyllene	0.3
caryophyllene oxide	0.3
cis-α-bisabolene	2.1
cis-β-farnesene	0.3
estragole	75.2
levomenthol	0.4
linalool	19.7
α-bergamotene	0.9
α-citral	0.7

	EO54
carvone	0.6
β-caryophyllene	1.8
cis-geraniol	76.5
cis-β-ocimene	0.3
farnesol	0.7
geranyl acetate	13.0
humulene	0.2
linalool	4.1
nerol acetate	1.0
α-citral	0.7
β-ocimene	1.1

	EO55
eucalyptol	53.9
eugenol	1.9
limonene	2.0
linalool	3.0
methyleugenol	4.2
myrtenol	0.3
o-cymene	2.4
sabinene	3.2
terpinen-4-ol	3.0
α-pinene	3.2
α-terpineol acetate	18.8
β-pinene	2.3
γ-terpinene	1.8

	EO56
anethole	88.5
β-caryophyllene	0.4
estragole	3.8
isohomogenol	0.2
limonene	1.6
linalool	0.9
nerolidol	0.3
p-acetonylanisole	0.9
p-anisaldehyde	2.1
terpinen-4-ol	0.3
α-bergamotene	0.5
α-pinene	0.3
α-terpineol	0.2

	EO57
3-carene	0.5
4-epi-cubebol	1.7
acetic acid	0.7
bornyl acetate	1.0
β-caryophyllene	9.1
caryophyllene oxide	3.0
cis-verbenol	1.8
humulene	2.0
humulene epoxide 2	1.1
limonene	20.3
myrtenyl acetate	0.3
o-cymene	3.7
p-cymen-8-ol	0.2
pinocarveol	0.9
sabinene	2.8
terpinen-4-ol	0.6
viridiflorol	0.5
α-copaene	2.3
α-cubebene	0.4
α-muurolene	0.4
α-phellandrene	2.0
α-pinene	27.0
α-selinene	1.1
α-terpineol	0.5
β-elemene	4.0
β-eudesmol	0.3
β-myrcene	3.0
β-phellandrene	1.0
β-pinene	1.0
β-selinene	1.6
γ-eudesmol	0.5
δ-cadinene	2.1
τ-cadinol	2.3

	EO58
.τ.-muurolol	0.1
calamenene	0.7
β-caryophyllene	7.3
caryophyllene oxide	0.2
cinerolon	4.6
cubenol	0.2
cyclohexanone, 2-(1-methylethylidene)-	1.0
epi-bicyclosesquiphellandrene	0.9
germacrene D	5.2
humulene	1.1
jasmone	0.6
limonene	8.8
myrtenal	0.3
pulegone	59.8
sabinene	0.6
terpinen-4-ol	0.7
thymol	1.2
α-cadinol	0.2
α-cubebene	0.5
α-gurjunene	0.4
α-muurolene	0.2
α-pinene	1.0
β-ocimene	0.1
β-pinene	3.8
γ-cadinene	0.2
γ-terpinene	0.1

	EO59
1-octen-3-ol	0.7
borneol	0.6
carvacrol	61.0
β-caryophyllene	13.6
caryophyllene oxide	1.4
cis-β-terpineol	0.2
humulene	0.6
limonene	0.3
linalool	1.8
p-cymene	7.1
thymol	0.5
α-citral	0.2
α-terpinene	1.5
α-thujene	1.0
β-bisabolene	1.6
β-myrcene	1.6
β-phellandrene	0.3
γ-terpinene	6.0

	EO60
1-octen-3-ol	0.6
borneol	2.5
camphene	0.6
carvacrol	0.7
β-caryophyllene	3.4
caryophyllene oxide	1.8
eucalyptol	0.9
isothymol methyl ether	6.0
limonene	0.4
linalool	3.7
methyl thymyl ether	10.7
p-cymene	30.5
thymol	34.1
α-pinene	0.9
α-terpineol	0.3
β-myrcene	1.0
γ-muurolene	0.3
γ-terpinene	1.8

	EO61
1-octen-3-ol	0.5
alcanfor	0.3
borneol	0.3
carvacrol	0.8
β-caryophyllene	2.9
caryophyllene oxide	0.6
eucalyptol	0.5
humulene	0.3
isothymol methyl ether	7.4
limonene	0.4
methyl thymyl ether	4.9
p-cymen-8-ol	0.1
p-cymene	18.5
thymol	34.7
thymol acetate	0.3
α-muurolene	0.2
α-phellandrene	0.2
α-terpineol	0.2
α-thujene	1.1
β-bisabolene	5.0
β-bourbonene	0.4
β-myrcene	1.3
γ-cadinene	0.4
γ-muurolene	0.6
γ-terpinene	17.1
δ-cadinene	0.7
